# The SMA Clinical Trial Readiness Program: creation and evaluation of a program to enhance SMA trial readiness in the United States

**DOI:** 10.1186/s13023-020-01387-8

**Published:** 2020-05-22

**Authors:** Ilse Peterson, Rosángel Cruz, Fatou Sarr, Ann Marie Stanley, Jill Jarecki

**Affiliations:** 1 Faegre Drinker Biddle & Reath LLP, Washington, DC USA; 2 Cure SMA, Elk Grove Village, IL USA

**Keywords:** Spinal muscular atrophy, Rare disease, Clinical trial coordination, Clinical trial readiness, Clinical trial best practices, Physical therapist and evaluator readiness, Rare disease clinical trial

## Abstract

Spinal muscular atrophy (SMA) is a rare neuromuscular disease with a rapidly evolving treatment landscape. To better meet the needs of trial sponsors and the patient community in the United States (US) in this evolving context, Cure SMA established a clinical trial readiness program for new and prospective SMA clinical trial sites. Program development was informed by a review of the SMA clinical trial landscape, successful NMD trial and care networks, and factors important to effective trial conduct in SMA. The program was piloted in 2018 with a virtual site readiness evaluation, a trial readiness toolkit, and a readiness program for physical therapists and clinical evaluators. Nine US research hospitals participated in the pilot. Cure SMA evaluated the pilot program and resources through feedback surveys, which supported the program’s relevance and value. Since 2018, the program has been expanded with additional sites, new best practices toolkits, and workshops. In partnership with Cure SMA, SMA Europe is also extending programming to European countries. The program is significant as an example of a patient advocacy group working successfully with pharmaceutical companies, other patient advocacy organizations, and research hospitals to promote trial readiness, and may serve as a model for organizations in other regions and diseases.

## Background

Spinal muscular atrophy (SMA) was historically the number one genetic cause of death for infants, with an estimated incidence between 1 in 10,000 to 11,000 live births [1, 2]. It is an autosomal recessive neuromuscular disease (NMD) caused by a homozygous deletion or mutation of the survival motor neuron 1 (*SMN1)* gene on chromosome 5q13, characterized by progressive muscle wasting and debilitating weakness [3–7]. The disease has been classified into subtypes based upon age of onset and motor function achieved [4, 8–10]. Type I has onset in infancy and is the most severe and common subtype (representing 50–60% of diagnoses), whereas types II, III, and IV represent later onset, milder phenotypes [4, 9–15].

Over the past two decades, understanding of SMA pathogenesis, natural history, and treatment pathways has evolved significantly. Major advances began with identification of the genes *SMN1* and *SMN2*, the latter of which is a partially functional analog whose copy number is inversely correlated with disease severity [3, 16]. Subsequent advances included development of mouse models; therapeutic target identification; emergence of new therapeutic modalities; natural history studies; development of standard of care; and establishment of reliable, sensitive, and meaningful outcome measures for SMA [4, 5, 7, 8, 15, 17–28]. These advances enabled therapeutic pipeline growth and paved the way for clinical trials in SMA, the first of which began in 2011 [5, 17, 29, 30]. In December 2016, the antisense oligonucleotide (ASO) nusinersen became the first FDA-approved drug for all types of SMA [31]. In May 2019, Zolgensma became the first FDA-approved gene therapy for SMA, indicated for children under two years [32]. As of the time of writing, eight programs were in phase I-III and open-label extension trials, and several others were in the preclinical phase [33, 34].

With these advances, new challenges and opportunities have emerged. The availability of therapeutics has heightened the importance of disease awareness and early diagnosis, as early intervention can significantly improve clinical outcomes [35]. The drug pipeline has also created a need for more trial sites to enable broader access to trials, prevent strain on existing sites, and conduct adult trials; increased the need for sites to be skilled in outcome measure assessments for trials and insurance renewals for approved drugs; raised considerations about access; and created a need for novel, clinically meaningful outcome measures to assess changes in an evolving, more chronic population [30, 33]. Finally, growing emphasis on the patient voice has led to expanded focus on understanding disease burden, meaningful outcomes, and patients’ and caregivers’ benefit-risk perspectives [36–41].

The SMA Industry Collaboration (IC) was established by Cure SMA in 2016 to address scientific, clinical, and regulatory challenges associated with SMA therapeutic development and evaluation. Cure SMA is dedicated to the treatment and cure of SMA, and the IC leverages the experience, expertise, and resources of Cure SMA, pharmaceutical companies, and other nonprofits to advance its goals. One of the IC’s priorities is to promote readiness for SMA clinical trials by ensuring trial sites have the capabilities and knowledge to run trials effectively. Trial readiness is critically important in SMA because these trials involve large, multidisciplinary teams, and managing a complex disease with potentially life-threatening severity and evolving phenotypes complicates trial management.

The Cure SMA Clinical Trial Readiness Program represents the first major initiative by Cure SMA and the IC to support SMA trial site readiness. This program provides US-based clinical research sites with resources to evaluate and optimize their readiness for SMA trials. Its ultimate goals are to alleviate challenges related to site capacity and trial access within the US, while sensitizing sites to the evolving needs of SMA patients and families. The program was piloted in 2018 and subsequently expanded. In collaboration with Cure SMA, SMA Europe is also extending program elements to Europe. The program holds significance for the SMA community globally as well as the broader rare disease community, as it may serve as a model for patient advocacy organizations in other regions and diseases.

### I. Landscape assessment: evaluating clinical trial needs and defining key attributes of site readiness for SMA trials

**Fig. 1:**
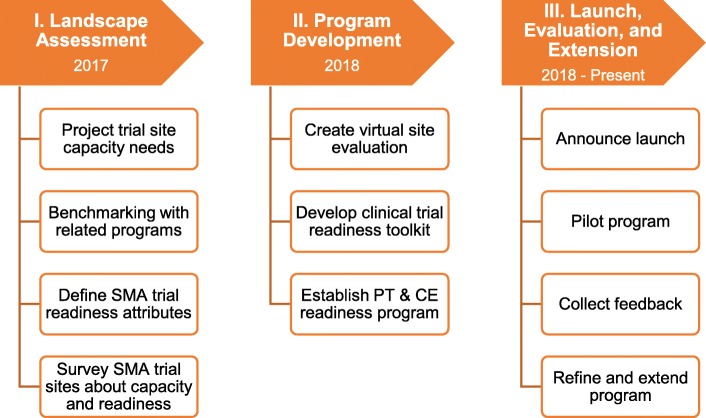
Establishment of the Cure SMA Clinical Trial Readiness Program. Establishment of this program included three phases. Prior to creating the Clinical Trial Readiness Program, Cure SMA sought insight into the need for new clinical trial sites, existing clinical trial and care readiness programs, and factors important to trial management in SMA. Findings from this assessment informed the scope and focus of the trial readiness program, which was developed in 2018 and – in its pilot phase – included three components: a virtual site evaluation, a program for physical therapists and clinical evaluators, and a clinical trial readiness toolkit. The program was launched in the spring of 2018, and has since been refined and expanded to include best practices workshops and toolkits for clinical research coordinators as well as physical therapists and clinical evaluators

Establishment of the Clinical Trial Readiness Program included three phases, beginning with a landscape assessment (see Fig. 1). This assessment focused on understanding the SMA clinical trial landscape, successful trial and care networks, and factors important to effective trial conduct in SMA. Its overarching goal was to help ensure that the trial readiness program would be responsive to the interests of sponsors, sites, and patients.

#### Projecting future SMA clinical trial recruitment and capacity needs

Cure SMA began the landscape assessment by estimating recruitment needs for SMA clinical trials from 2011 through 2022, to confirm the need for new clinical trial sites and the Clinical Trial Readiness Program. Using information from ClinicalTrials.gov and the SMA drug pipeline, Cure SMA concluded that to meet recruitment targets for the next five years (2018–2022), existing SMA clinical trial sites would need to recruit approximately twice as many trial participants as they had in the past.^1^ (For details, see Additional file 1.) Cure SMA anticipated that this could strain sites and be difficult because of the geographic distribution of patients. This led to the conclusion that new clinical trial sites–and this program–were needed.

#### Learning from existing clinical trial and care networks

Cure SMA’s second step was to learn about related programs in other NMDs that could inform creation of the trial readiness program. Through semi-structured interviews with leaders of three established research and care networks, Cure SMA identified several common elements of successful programs (see Table 1). These would inform the 2017 survey of experienced trial sites described below and the readiness program itself (see Tables 2 and 3).

**Table 1:** Common elements of successful NMD research and care networks

Through benchmarking with leaders of established neuromuscular disease research and care networks–including NeuroNEXT, the Parent Project Muscular Dystrophy (PPMD) Certified Duchenne Care Center Program (CDCCP), and the Cooperative International Neuromuscular Research Group (CINRG)–Cure SMA identified several common elements of successful programs. These elements–and how they informed Cure SMA’s approach to creating the Clinical Trial Readiness Program–are described below.	
Recommendations from Established Programs & Actions Taken by Cure SMA	
**Consider human and financial resources needed to make programs sustainable** ▪ Proactive and detailed approach to project planning and budgeting at outset, followed by annual strategic planning and budgeting ▪ Virtual activities enable greater sustainability **Understand established sites’ needs and research infrastructure, staffing, capabilities, and ability to coordinate care** ▪ 2017 survey of SMA clinical trial sites provided insight on established sites ▪ Similar information addressed in site evaluation survey **Develop baseline criteria for participation** ▪ Baseline criteria identified based on consensus readiness checklist and sponsor input **Ensure evaluations are sensitive to site workloads** ▪ Virtual site evaluation focused on elements identified as important by sites and sponsors **Establish site “champions” to spearhead readiness efforts and manage trials** ▪ Direct engagement of principal investigators (PIs) who can drive internal decision-making **Consider timelines and enrollment targets for future trials** ▪ 2017 recruitment model projected future needs ▪ Ongoing review of planned trials and discussion with sponsors about anticipated needs

**Table 2:** Recommended actions to enhance site readiness for SMA clinical trials

As part of the readiness program, Cure SMA developed a list of recommended steps sites can take to support readiness for SMA clinical trials. These were based on benchmarking with other organizations, the 2017 survey of experienced research sites, and input from experienced trial practitioners. They were shared with program participants during site interviews and are also reflected in program toolkits.	
Recommendations for Clinical Trial Sites	
**Optimize site infrastructure and logistics to accommodate patient needs** ▪ Collocate assessments, procedures, and dosing to reduce the stress of research visits for patients and families and streamline the flow of visits for research teams. ▪ Offer resources to support families that travel together – such as play rooms for siblings – to reduce stress for parents. **Familiarize staff who support clinical trials with SMA and how it can affect patients and families** ▪ Provide opportunities for team members to learn about SMA. This can promote patient-centered trial management and help them anticipate and prepare to address challenges that may arise during trials. ▪ Allow newer team members to shadow experienced colleagues to learn about patients and protocols. **Take proactive steps to promote strong team coordination** ▪ Streamline operations by clearly delineating responsibilities, communicating frequently, and using checklists and scheduling tools. ▪ Use weekly clinic and research meetings to keep teams on the same page about matters pertinent to trial conduct. **Use checklists, templates, and SOPs to aid trial management** ▪ Create and implement checklists, templates, and standard operating procedures (SOPs) to promote prompt and thorough completion of requisite activities. ▪ Use checklists and templates for important communications, to make sure patients and team members receive the information that they need when they need it. **Share information in a patient-centric way and be open to learning from patients and families** ▪ Be attentive to how information is shared with patients and families. Communicate clearly and concisely, being mindful about word choice and understanding. ▪ Provide opportunities for questions and clarification of information, and be willing to listen to and learn from patients and families.

**Table 3:** Characteristics of Sites that Participated in 2018 Readiness Program Pilot (*N* = 9)

Characteristics^a^	# of Sites with	% of Sites with
**Minimum Criteria**		
Clinical research infrastructure	9	100%
Seeing SMA patients for research or care	9	100%
**Site Research Capabilities and Experience**		
Dedicated clinical research unit	8	89%
Clinical trial experience (any)	9	100%
Neuromuscular clinical trial experience	5	56%
Conducting other SMA research studies (not clinical trials)	4	44%
Active enrollment for other SMA research studies (not clinical trials)	3	33%
**Patient Population**		
Children	8	89%
Adults	6	67%
**Principal Investigator Experience**		
SMA clinical trial experience	5	56%
Other neuromuscular clinical trial experience (outside of SMA)	7	78%
Certified Principal Investigator	0	0%
**Clinical Research Coordinator Experience**		
SMA clinical trial experience	2	22%
Other neuromuscular clinical trial experience (outside of SMA)	5	56%
Coordinator(s) has completed ACRP CRC or SOCRA certification	6	67%
**Physical Therapist Experience**		
SMA-specific motor function outcome measures (clinical evaluation)	9	100%
SMA-specific motor function outcome measures (clinical trials)	5	56%
Other neuromuscular disease outcome measures (not SMA)	8	89%
Completed reliability training for motor function outcome measures	8	89%
**Staff Training Related to Conduct of Clinical Research**		
Staff involved in clinical research have conducted all or majority of the listed training programs	9	100%
**Clinical Trial Operations**		
IRB (Centralized or Local)	9	100%
Well-documented informed consent process	9	100%
Established, well-documented approach for ensuring adherence to study protocol	9	100%
Established and well-documented approach to PI oversight	9	100%

^a^The characteristics presented in this table are those that were included in the site readiness checklist

#### Identifying critical elements of site readiness for SMA clinical trials

Next, Cure SMA worked with the IC to build a consensus readiness checklist for SMA clinical trials. This checklist focused on essential factors relating to site and team experience in SMA and NMDs; care coordination; and site infrastructure, operations, and compliance. It formed the basis for the survey of experienced SMA trial sites and the readiness program’s trial site evaluation (see Additional files 2, 3 and 4 and Table 3).

#### Understanding the experiences and capacity of established SMA trial sites

**Fig. 2:**
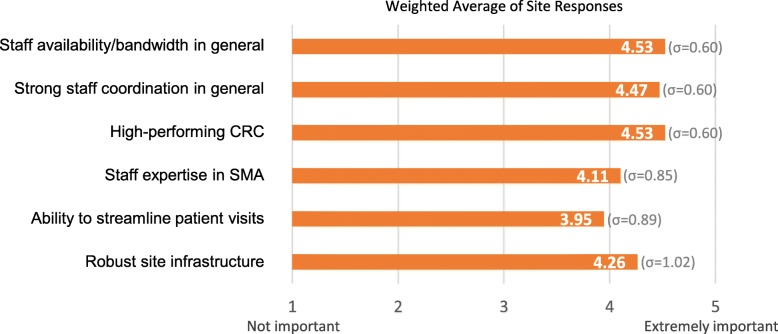
Factors important to successful clinical trial management in SMA. Results of a 2017 survey of US-based SMA clinical trial sites played a critical role in informing Cure SMA’s approach to creating the Clinical Trial Readiness Program. Weighted averages of responses (*n* = 19) to the question “How important have the following factors been in enabling you to successfully run clinical trials in SMA?” revealed that, overall, sites viewed staff bandwidth and a high-performing clinical trial coordinator as most important to successful trial management, closely followed by strong staff coordination. The rating scale for this question included the options: not important, somewhat important, important, very important, and extremely important. These findings contributed to Cure SMA’s decisions to create resources on trial coordination and SMA as well as materials specifically for clinical research coordinators. They also informed recommendations made during site interviews in 2018

**Fig. 3:**
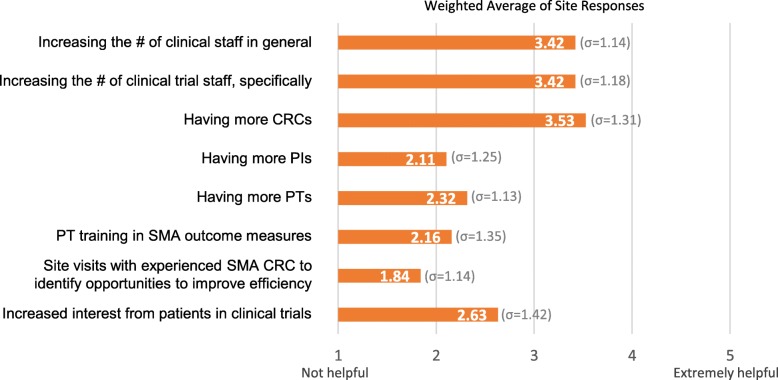
Factors that could help sites to take on new clinical trials in SMA. Weighted averages of responses to the question “How helpful would the following be in increasing your ability to take on additional SMA trials?” in Cure SMA’s 2017 survey of US-based SMA clinical trial sites (n = 19) indicated that sites overall would find increased numbers of personnel most helpful. The rating scale for this question included the options: not helpful, somewhat helpful, helpful, very helpful, and extremely helpful. Although Cure SMA has not able to directly support staff increases as part of the Clinical Trial Readiness Program, program resources have focused on promoting efficient and effective team operations

The final landscape assessment activity was to survey experienced US-based SMA trial sites about factors enabling successful trial management, how Cure SMA could support trial readiness, and their capacity for future trials. In August 2017, Cure SMA invited all US sites that had conducted SMA trials (*N* = 21) to participate in this survey, which nineteen sites completed (*n* = 19). Results confirmed the importance of several factors to effective trial management: staff bandwidth and coordination, CRCs, expertise in SMA, and site infrastructure (see Figs. 2-3 and Additional file 2). These findings shaped the focus and content of the readiness program activities and materials, spurring Cure SMA to focus on supporting SMA expertise and best practices for trial conduct. Results also indicated that established sites had some capacity for additional trials.

### II. Program development: creating resources to support trial site readiness

The Cure SMA Clinical Trial Readiness Program was developed in 2018. In its pilot phase, the program included: (1) an evaluation addressing overall site readiness, which was informed by the consensus readiness checklist and 2017 survey; (2) a Clinical Trial Readiness Toolkit; and (3) a readiness program for physical therapists (PTs) and clinical evaluators (CEs). Together, these components were intended to empower research teams with knowledge about SMA and strategies to support effective, patient-focused trial management.

#### Component 1: trial site readiness evaluation

The first component of the pilot program was an educational trial site readiness evaluation. To be sustainable and sensitive to site workloads, this was formatted as a virtual assessment with two sequential online surveys (see Additional file 3) and a phone interview.^2^ The surveys evaluated baseline criteria for trial readiness, including experience with SMA patients in clinical and research settings; clinical trial experience; and the experience of individual principal investigators (PIs), PTs, and CRCs. The phone interviews focused on strategies to prepare for trials and promote patient-centric trial coordination (see Table 2). After the interviews, sites received copies of their in-depth survey and a checklist reflecting their experience and capabilities. With site permission, the checklists were shared with IC participants (see Additional file 5). Finally, sites were granted access to the Cure SMA Clinical Trial Readiness Toolkit and invited to participate in the PT and CE Readiness Program.

#### Component 2: Clinical Trial Readiness Toolkit & Clinical Research Coordinator Best Practices

The Clinical Trial Readiness Toolkit is an in-depth document for research teams with practical information on SMA and effective, patient-centered trial conduct [42]. It was developed with extensive input from experienced PIs, CRCs, and industry experts, and addresses the basics of SMA, the therapeutic landscape, the clinical trials process, and external educational resources. The original version also included a dedicated appendix on best practices for CRCs.

#### Component 3: physical therapist and clinical evaluator readiness program

Cure SMA’s PT and CE Readiness Program was created because of the importance of PTs and CEs in assessing patient progress toward trial endpoints. The program was developed with leadership from Cure SMA and two PTs with SMA expertise. PT and CE participants begin the program by completing a questionnaire about their experience evaluating patients in NMDs, SMA, and in clinical and research settings. Participants then receive tailored recommendations on preparing for SMA trials, and are asked for input on how Cure SMA could further support their development. During the pilot, participants were also invited to comment on an outline for a PT toolkit that would be launched in 2020.

### III. Program pilot, evaluation, and extension

#### Launch of the pilot program

The trial readiness program pilot was announced in March 2018 during a webinar on Cure SMA’s Clinical Care Network, Clinical Data Registry, and readiness program. Representatives from 142 healthcare, industry, and nonprofit organizations were invited, and 62 individuals attended. Interested sites meeting the criteria for participation–which included experience with clinical trials and seeing SMA patients–were invited to contact Cure SMA to participate. Between April 2018 and January 2019, nine sites expressed interest in and completed the program. These sites had a broad range of experience in neuromuscular and SMA clinical trials. All had experience with SMA patients in clinical contexts (see Table 3).

#### Evaluation of the pilot program

Effectiveness of the program pilot was assessed with feedback surveys, deemed the most feasible approach given resource and site bandwidth constraints. Responses to a program survey completed by five pilot sites indicated that the program had helped all respondents in some way (e.g. by helping to identify steps to enhance trial readiness, or learning specific trial management tactics for use in day-to-day operations); all respondents also reported using the toolkit to onboard team members at their sites. In addition, responses to a toolkit feedback survey shared with readiness sites and CRCs who provided input for the CRC best practices confirmed that the toolkit could be incorporated into clinical practice, increased understanding of SMA and clinical research concepts, and generated awareness of potential challenges in SMA clinical trials as well as strategies for addressing challenges; this survey was completed by four pilot site PIs and one CRC. Feedback from IC members further validated the program’s utility in establishing a common baseline for trial site readiness and providing greater awareness about sites interested in SMA trials.

#### Program extension

Based on the positive feedback received during the pilot, Cure SMA has refined and extended this program. Extension is focusing on addressing areas of high need, by engaging sites that are in geographic regions without SMA trial sites and that see adults. In 2019, four new sites completed the site readiness evaluation, which was streamlined to one survey (Additional file 4).^3^ Additional areas of focus include new educational resources and PT readiness. Cure SMA has launched best practices workshops for CRCs and PTs/CEs, first held at the June 2019 Cure SMA Annual Conference and attended by 16 CRCs and 17 PTs; expanded the CRC best practices from the original toolkit into a standalone document; and launched Best Practices for Physical Therapists and Clinical Evaluators in SMA [43, 44]. The latter contains comprehensive information on the role and responsibilities of PTs/CEs; outcome measures used in SMA; SMA trial preparation; and other topics such as standard of care and supportive care. Finally, Cure SMA has made program resources globally accessible. In late 2019, Cure SMA launched a new webpage with program information and free digital copies of all toolkits (www.curesma.org/clinical-trial-readiness) [45]. In partnership with SMA Europe, program components are also being extended to Europe. SMA Europe has surveyed European centers about capacity and needs, planned best practices workshops, and is adapting and translating the toolkits for European sites.

## Conclusions

Cure SMA’s Clinical Trial Readiness Program represents a novel effort to support clinical trial site readiness for a rare disease. While other organizations have developed related programs, this is a unique example of a patient advocacy group establishing a program in a collaborative setting with pharmaceutical sponsors, other patient advocacy organizations, and research hospitals. This program has created a new means for engaging research sites interested in SMA trials, providing tangible resources to optimize readiness, promote patient-centered trial management, and increase sites’ visibility with sponsors. To ensure sustained relevance, Cure SMA will adapt the program to the needs of sponsors and sites over time.

Creation of this program has brought several key opportunities and challenges surrounding clinical trials in rare diseases to the forefront. On one hand, because the number of companies and PIs involved in clinical research was relatively small, Cure SMA could obtain input from all companies and nearly all PIs active in US SMA clinical trials while building this program. This provided a more holistic view of the SMA trial landscape and community needs. On the other hand, the relatively small number of clinician researchers with SMA expertise will make identification of prospective readiness sites increasingly challenging as the program expands. As a result, Cure SMA will need to determine how to balance the need for in-depth expertise with the desire to expand the geographic reach of SMA trials.

This experience also highlighted potential needs and limitations of clinical research sites working on complex rare disease trials. Interactions with sites made it clear that even when PIs have interest and expertise, finding internal resources and building the right team for SMA trials may not be easy. Even when a team exists, preparing that team for work in a specific rare disease can be challenging because of bandwidth and resource constraints. This underscores the importance of disease-specific training materials that support effective, patient-focused trial management. While Cure SMA’s resources help to fill a gap for SMA clinical trials, resources for learning about rare diseases and unique considerations for managing trials within these diseases often do not exist.

Lastly, it is important to note a few limitations of this program. First, although Cure SMA has provided trial sites with new resources that support optimized trial readiness and patient-centered management, it remains incumbent upon PIs, CRCs, and PT/CEs to utilize these resources and implement changes in their practices. Second, while program outcomes were assessed via surveys, objective metrics would provide more definitive information on outcomes and impact. Use of such metrics was not feasible given resource constraints and concerns about burdening sites, but represents an area for future exploration. Third, it should be noted that the sites which participated in this program were those with intrinsic interest in operational effectiveness and patient-centered care, and Cure SMA was not able to gain detailed information about why other sites have not participated apart from feedback about limited bandwidth. This could be further explored as the program matures.

With this program, Cure SMA hopes to provide a roadmap for how other rare disease communities might partner with industry and clinical research sites to evaluate and optimize clinical trial readiness for their diseases. This model could be replicated for other diseases and in other regions, providing a potentially efficient way to support clinical trial readiness as more rare disease trials take place. Particularly for patient communities that have forged links with industry and academic researchers, this model will be relatively quick to build and scale; it may also be more sustainable to operate than a more formal trial network, while still providing value to research sites. Finally, in addition to supporting trial readiness generally, implementation of this model has potential to amplify the patient voice and promote more patient-centric clinical trial management, by sensitizing sites to unique needs of specific patient communities.

## Supplementary information

**Additional file 1.** Description of methodology for trial landscape review.

**Additional file 2.** Results of the 2017 SMA clinical trial site survey with survey tool.

**Additional file 3.** Site evaluation questionnaires used in 2018.

**Additional file 4.** Streamlined site evaluation questionnaire used in 2019.

**Additional file 5.** Site readiness checklist.

## Data Availability

The data regarding SMA phase I-III trials occurring from 2011 to 2017 that supported the findings of this study are available on Clinicaltrials.gov with the identifiers [NCT01494701; NCT01703988; NCT01780246; NCT02052791; NCT02193074; NCT02292537; NCT02240355; NCT01302600; NCT02268552; NCT01839656; NCT02462759; NCT02594124; NCT02386553; NCT02122952; NCT02644668; NCT02628743; NCT03032172; NCT02913482; NCT02908685]. Readers may contact Cure SMA for additional details on the assessment of the 2011–2017 trial landscape and projection of 2018–2022 trial needs. Datasets from the 2017 survey and site readiness program are not available because Cure SMA does not have consent to publish them.

## Abbreviations

ASO: Antisense oligonucleotide
CDCCP: Parent Project Muscular Dystrophy Certified Duchenne Care Center Program
CE: Clinical evaluator
CINRG: Cooperative International Neuromuscular Research Group
CRC: Clinical research coordinator
IC: Cure SMA Industry Collaboration
MFM: Motor function measure
NMD: Neuromuscular disorders
PI: Principal investigator
PT: Physical therapist
SMA: Spinal muscular atrophy
SMN: Survival motor neuron
SOP: Standard operating procedure
US: United States

## Footnotes

1 The primary goals of this work were to obtain order-of-magnitude estimates of (1) average numbers of trial participants at trial sites within and outside of the United States and (2) future recruitment targets. To do this, Cure SMA used information from Clinicaltrials.gov to calculate average numbers of trial participants at SMA clinical trial sites globally. Based on the drug pipeline, Cure SMA then projected anticipated recruitment needs for 2018–2022. The purpose was not to create a highly precise model, but to obtain practical information that could confirm the perceived need for future trial sites. For a more detailed description of methodology, please see Additional file 1.

2 Importantly, while the program presented here is intended to be useful for both sites and sponsors, it was not intended to be a research network per se, nor to replace site initiation visits conducted prior to a trial.

3 In 2019, thirteen new sites were contacted directly about participation in the program. Information was also shared publicly at the Cure SMA Annual Conference. Four of the thirteen sites that were contacted directly completed the program in 2019, two deferred participation to 2020 due to bandwidth constraints, one indicated interest but did not complete the program, and seven did not respond. Additional sites will be invited to participate in 2020.
