# Supplementary material for: The SMA Clinical Trial Readiness Program: creation and evaluation of a program to enhance SMA trial readiness in the United States
Source: Orphanet J Rare Dis. 2020 May 22;15:118. doi: 10.1186/s13023-020-01387-8 (PMC7564894; doi:10.1186/s13023-020-01387-8)
Supplement: Supplementary file 1 — Additional file 1. Description of methodology for trial landscape review. [file 13023_2020_1387_MOESM1_ESM.pdf]

Additional File 1. Methodology for Assessing 2011-2017 SMA Clinical Trial Landscape and Projecting 2018-2022 Recruitment Needs

Prior to creating the Clinical Trial Readiness Program, Cure SMA reviewed the SMA clinical trial landscape. The primary goals of this review, which was conducted in May 2017, were to establish order-of-magnitude estimates of:

(1) average numbers of trial participants at trial sites globally, and
(2) enrollment targets for trials planned over the next five years.

This review was critical to understanding the degree to which new trial sites would be needed to support future trials. Cure SMA's methodology for this review was as follows.

Collection of Information

From Clinicaltrials.gov, Cure SMA obtained information about numbers and types of SMA trials completed, ongoing, or planned, as well as trial sites and recruitment targets for each trial. This information was downloaded in May 2017. A sample of information collected and used in calculations is presented in Table 1 below. Twenty trials were listed on Clinicaltrials.gov at the time.

Table 1. Snapshot of information used to assess 2011-2017 SMA clinical trial landscape*

Study	Projected enrollment	Phase	Starts	Recruiting	# Sites	Trial status
ISIS 396443- CS1	28	I	2011	Types II, III 2-14	4 sites (US only)	Completed; rolled over to CS2
ISIS 396443-CS2/CS10	52	I/II	2012	Types II, III 2-14	4 sites (US only)	Completed; rolled over to CS12
ISIS 396443- CS12	52	II	2013	Types II, III 2-15	4 sites (US only)	Completed; rolled over to SHINE
ISIS 396443-CS3B (ENDEAR)	122	III	2014	Type I, SHAM <7 months	31 sites (12 US)	Completed; rolled over to SHINE
ISIS 396443-CS4 (CHERISH)	126	III	2014	Type III, SHAM 2-12	24 sites (11 US)	Completed; rolled over to SHINE
TRO19622	165	II	2011	Types I-III, 3-25	25 (Europe only)	Completed – rolled over to OLE
AVXS-101	15	I/II	2014	Type I, Up to 6 months	1 (US)	Ongoing 2030

*This table represents a subset of information collected from Clinicaltrials.gov for the purposes of this assessment.

Estimation of Mean Trial Participants Across Sites for Completed and Ongoing Trials

Cure SMA estimated mean numbers of trial participants across sites by totaling the numbers of trials, sites, and recruitment targets; adjusting for rollover of patients in sequential trials and terminated studies (which were excluded from calculation); and dividing recruitment targets by numbers of unique sites engaged in trials. Cure SMA reviewed information on trials that had been completed and were ongoing at the time of the assessment separately. As the goal of this

work to obtain order-of-magnitude estimates that could inform program development and goals, simple means were used in calculations. Results of these calculations are presented below.

Table 2. Recruitment estimates for clinical trials completed as of May 2017*

	Biogen, ENDEAR	Biogen, CHERISH	Biogen, ISIS CS1	Biogen, ISIS CS2	Biogen, ISIS CS10	Biogen, ISIS CS12	Roche, Olesoxime (TRO19622)	Totals	
**Number of Trial Sites**									
Trial sites globally	31	24	4	4	4	4	17	51	Unique sites
US trial sites	12	11	4	4	4	4	0	13	Unique sites
Non-US trial sites	19	13	0	0	0	0	17	51	Unique sites
**Recruitment Targets**									
Global recruitment target	122	126	28	52	52	52	165	300	Global total**
US recruitment target	47	58	28	52	52	52	0	157	US total**
Recruitment by non-US sites	75	68	0	0	0	0	165	143	Non-US total**
**Mean Participants Per Site by Trial**									
Mean participants per site	3.9	5.3	7.0	13.0	13.0	13.0	9.7		
**Estimated Mean Participants Per Clinical Trial Site (All Trials)**									
Overall global mean	5.9	participants							
US trial sites	12.1	participants							
Non-US trial sites	2.8	participants							

* Estimates assume even distribution of patients across sites participating in trials for simplicity.
**Participants in ISIS trials rolled over into subsequent trials and were therefore only counted once when counting total numbers of participants.

Table 3. Recruitment estimates for clinical trials ongoing in May 2017*

	Biogen, NURTURE	Biogen, SHINE (ISIS CS11)	Biogen, ISIS CS3A	Biogen, EMBRACE	AxeXis AVX101	Cytokinetics; (Cohorts 1,2)	Roche, Olesoxime (TRO19622)	Totals	
**Number of Trial Sites**									
Trial sites globally	15	35	4	7	1	15	25	70	Unique sites
US trial sites	7	13	3	6	1	11	0	21	Unique sites
Non-US trial sites	8	22	1	1	0	4	25	49	Unique sites
**Recruitment Targets**									
Global recruitment target	25	289	20	21	15	36	165	571	Global total
US recruitment target	12	107	15	18	15	26	0	193	US total
Recruitment by non-US sites	13	182	5	3	0	10	165	378	Non-US total
**Mean Participants Per Site by Trial**									
Mean participants per site	1.7	8.3	5.0	3.0	15.0	2.4	6.6		
**Estimated Mean Participants Per Clinical Trial Site (All Trials)**									
Overall global mean	8.2	participants							
US trial sites	9.2	participants							
Non-US trial sites	7.7	participants							

* Estimates assume even distribution of patients across sites participating in trials for simplicity.

Projection of Future Recruitment Targets

Recruitment targets for trials expected to enroll during 2018 – 2022 were estimated using the SMA drug pipeline. Cure SMA assumed that all programs active in 2017 would move forward. It was also assumed that recruitment targets for future phase I, II, and III trials would be similar in order-of-magnitude to past SMA trials within corresponding phases. Based on these

assumptions, Cure SMA projected that the total number of trial participants at sites participating in trials between 2018-2022 would be close to 1100, raising the estimated overall global average for numbers of participants per site during that time period to about 15 (for 73 established trial sites).

Conclusions

Comparing estimates for overall global averages of participants at trial sites for (1) ongoing and completed trials with (2) estimates for trials expected to enroll between 2018-2022, Cure SMA concluded that the number of trial participants at sites would need to roughly double to meet future recruitment targets if the number of trial sites remained constant. Given assumptions that such significant increases may strain established sites as well as considerations related to geographic distribution and the importance of increasing access to trials, it was concluded that a significant number of new clinical trials sites would be needed for future trials.
