# Supplementary material for: The SMA Clinical Trial Readiness Program: creation and evaluation of a program to enhance SMA trial readiness in the United States
Source: Orphanet J Rare Dis. 2020 May 22;15:118. doi: 10.1186/s13023-020-01387-8 (PMC7564894; doi:10.1186/s13023-020-01387-8)
Supplement: Supplementary file 2 — Additional file 2. Results of the 2017 SMA clinical trial site survey with survey tool. [file 13023_2020_1387_MOESM2_ESM.pdf]

Additional File 2. Results of the 2017 SMA Clinical Trial Site Survey

Background and Purpose of Survey

During August – September 2017, Cure SMA surveyed clinical trial sites with experience conducting clinical trials in SMA to (a) understand sites' capacity for future clinical trials in SMA, (b) identify factors that may limit site trial capacity and/or readiness, and (c) identify opportunities to increase capacity. All sites in the United States with experience conducting clinical trials (N=21) were contacted. Nineteen (n=19) sites completed the survey; one (1) site did not respond to the survey requests, and one (1) site declined to complete the survey. Following initial review of survey responses for completeness, a subset of sites was contacted to address outstanding questions about their submissions. Survey findings are presented here and have not been published prior to this.

Summary of Findings

On the whole, sites felt more strongly about the factors that enabled them to run trials than factors limiting their capacity. Key observations on specific factors are discussed below.

Patient Populations

The participation in research generally increased proportionally with total population size, although some sites had comparatively high levels of participation, while others were below average. Both the linear trend and numerical average suggest that clinical sites enroll on average approximately 20-25% of their total clinic and research population in clinical trials.

Studies Underway at Sites

There were large variations in the number of studies underway at different sites. There was only a weak correlation between the number of studies and the size of the SMA research population at individual sites and no clear relationship between the number of trials at a site and the percentage of the SMA patient population participating in research. Sites were not asked to list each of the trials underway to minimize burden; however we recognize that some sites only included trials underway while others included prospective trials (with IRB approval but no recruited patients), as part of their overall numbers.

Site Staff

There was large variability in staffing across sites, and no clear trends were identified when comparing the numbers of the key staff to the sizes of research population at SMA sites or the number of SMA studies underway.

Site Capacity

Notably, all but one of the survey respondents indicated that their sites have capacity to conduct additional SMA trials at this time.

Factors that Have Enabled Sites to Successfully Run Trials

Sites were asked to rank the significance of enabling factors including (a) clinical staff bandwidth, (b) staff and site coordination, (c) performance of the clinical trials coordinator, (d) staff expertise in SMA, and (e) site infrastructure. Staff bandwidth, coordination, and

performance of coordinators were all endorsed as extremely important to successful trial management, but no one factor clearly stood out as most important to successfully running trials.

Factors Preventing Sites from Taking on Additional Trials

Sites were asked to rank the significance of two categories of factors related to (a) the site and its staff and (b) patients. Potential site and staff limiting factors covered by the survey were clinical staff bandwidth, trial staff bandwidth (including bandwidth of staff with SMA experience specifically), infrastructure, and other factors. Bandwidth of clinical and trial staff emerged as most significant. However, these were only highly significant for a fraction of sites. They were not significant for about half of the sites. Bandwidth of staff with SMA expertise and limited site infrastructure did not appear to be significant factors. In terms of patient-related factors, sites were asked about patient interest, distance from sites, patient saturation due to ongoing open label extension trials, and increased numbers of patients being followed as new treatment options become available. No individual factors emerged as highly significant in limiting capacity.

Opportunities for Cure SMA & the Cure SMA Industry Collaboration to Support Sites

Sites were asked to rank a series of potential approaches to addressing capacity challenges, and to provide free text suggestions for activities Cure SMA could undertake to support sites. Items ranked by sites included increasing numbers of clinical staff, trial staff, coordinators, principal investigators, and physical therapists; PT training in outcome measures; site visits with an experienced trial coordinator to identify opportunities to improve efficiency; and increased patient interest in trials. Increasing numbers of clinical staff, trial staff, and coordinators would be most helpful to sites. PT training in outcome measures and visits from coordinators would be helpful to a handful of sites, but about half of the respondents indicated that PT training would not be helpful for their sites. In free-text responses, sites also suggested: raising awareness of trials amongst the patient community in general, raising awareness about under-utilized sites and assistance with enrollment, and grants to support expanded infrastructure and coordinators.

Application of Learnings

Learnings from this survey informed Cure SMA's plan for optimizing trial site readiness and capacity, and development of the Cure SMA Clinical Trial Readiness Program. Based on the initial findings of this survey, Cure SMA concluded that sites with experience running SMA trials may benefit most from awareness-raising communications about clinical trials generally; a subset of these sites may also benefit from resources to help coordinators optimize internal coordination and efficiency. Based on the factors respondents identified as most important to successfully running trials, Cure SMA concluded that resources likely to be of the greatest value to new SMA trial sites would include training and resources for clinical research coordinators and physical therapists, resources to support research teams' understanding of SMA and team coordination.

Results of Survey Analysis

Results of the analysis of survey data are presented below.

I. Patient Populations

The first section of the survey asked sites for information about their patient populations, including sizes of populations, participation in research, and factors driving referrals to the site.

Sizes of Sites' Patient Populations

When looking at the information obtained about patient populations – presented in the tables and charts below – it is clear that there is wide variation across sites. In terms of numbers of patients seen for routine clinical care, population sizes ranged from 10 to 200 patients, with an average size of 71.8 patients. Clinical trial population sizes ranged from two to 45, with an average size of 17 patients. Total patient population sizes (including patients seen for routine care, research and care, and research only) ranged from 14 to 215 patients, with an average size of 78.3. Only one site had a population greater than 150 patients.

Table 1. Sizes of Clinic, Research, and Total Patient Populations at Sites Surveyed (n=19)

	Minimum	Maximum	Median	Mean	St Dev
# Patients Receiving Routine Care	10	200	70	71.8	44.9
# Patients Seen for Clinical Trials	2	45	15	17.4	13.0
Total # Patients Seen (Clinical and Research)	14	215	73	78.3	47.8

To better understand the distribution of total patient and research population sizes across sites, sites were grouped into the size categories below. From these distributions, it is clear that there is greater variation in the sizes of research populations across sites than in the sizes of total populations.

Figure 1. Sizes of Patient Population Across Sites (n=19)

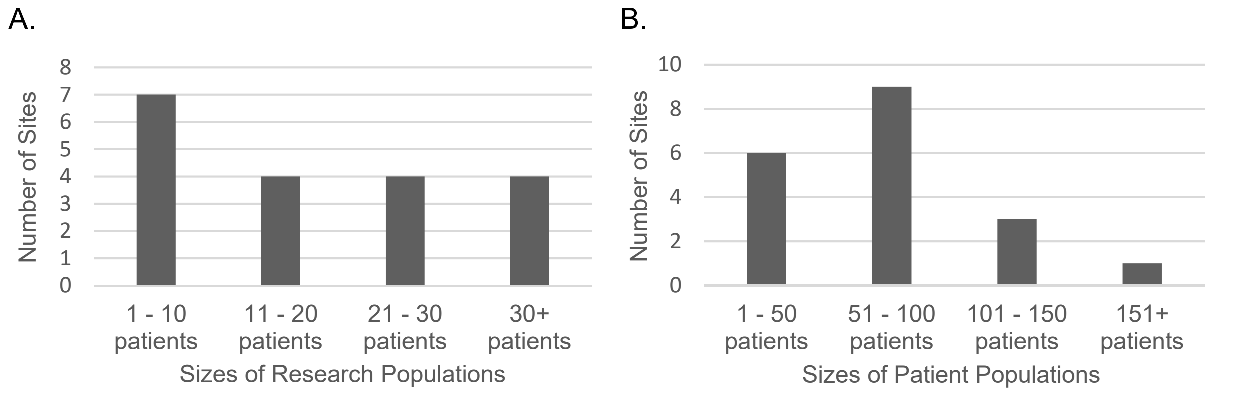

Figure A depicts the sizes of the research population across sites. Figure B depicts the sizes of the total patient (clinic and research) population across sites.

Participation in Research

As with population sizes, there was considerable variation in the percentages of total SMA patient populations participating in research across sites. The lowest participation rate was about 5%, while one site reported that 75% of its patients were involved in research (however, this was a unit with a very specific focus, which likely resulted in a higher rate of participation than most sites would otherwise have).

To identify potential trends in terms of research participation, research populations and the percent of total patient populations participating in research were plotted against total site populations. The former plot demonstrated that participation in research generally increased proportionally with total population size, although some sites were positive outliers (with comparatively high levels of participation), while others were below average.

Table 2. % of Patient Population Participating in Research Across Sites (n=19)

	Minimum	Maximum	Mean	St Dev
% of Patient Population Participating in Research	4.8%	75.0%	23.9%	17.7%

Figure 2. Research Participation at Sites (n=19)

A. B.

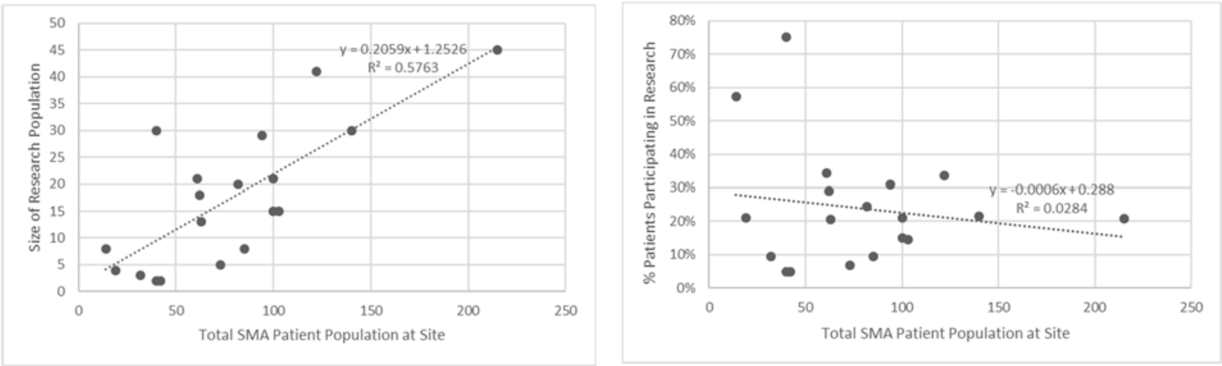

Figure A depicts the research population vs the total SMA patient population. Figure B depicts the percentage of the total population in research vs the total SMA patient population.

Ages of Patients Seen by Sites

While five of the sites surveyed only saw children, most saw both children and adults. Minimum and maximum ages seen within these categories are presented below.

Table 3. Ages of Patients Seen by Neurology Units (in Years)

	n	Youngest Age	Oldest Age
For sites that see children only	5	0	21
For sites that see adults only	0	N/A	N/A
For sites that see children and adults	14	3	100

Sources of Referrals

Respondents were asked how important particular factors were in driving referrals of SMA patients to their clinical trial site. SMA expertise and active clinical trials at the receiving site were the most strongly endorsed factors contributing to referrals, but lack of specialty care and capacity at the referring site were endorsed as important factors as well.

Figure 3. Factors Affecting Patient Referrals (n=19)

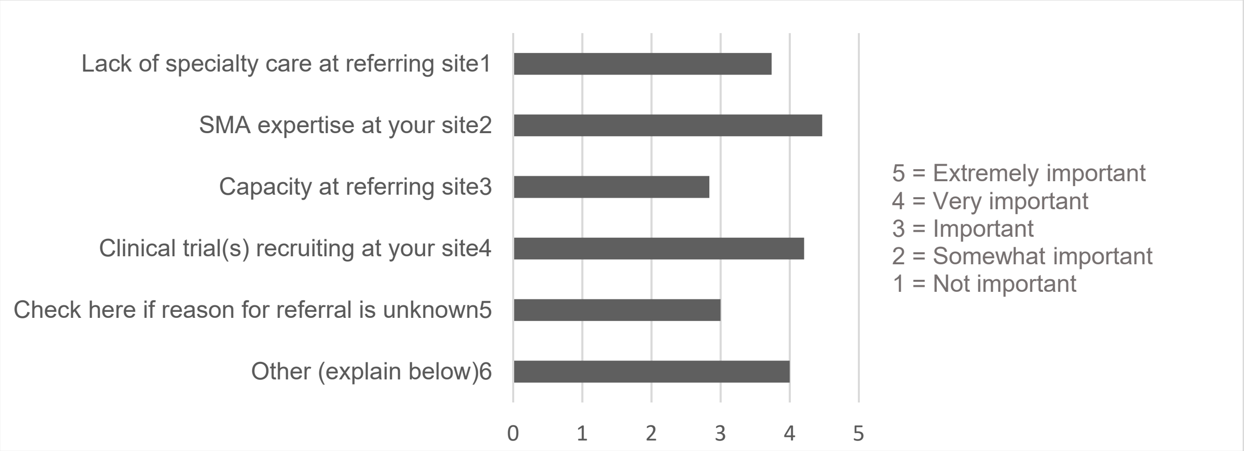

Participants were asked "How important have the following been in driving referrals to SMA patients to your site?"

1. Lack of specialty care at referring site (weighted average (WA) = 3.74)
2. SMA expertise at your site (WA = 4.47)
3. Capacity at referring site (WA = 2.84)
4. Clinical trial(s) recruiting at your site (WA = 4.21)
5. Check here if reason for referral is unknown (WA = 3)
6. Other (explain below) (WA = 4)

II. Studies Underway at Sites Surveyed

In the second major section of the survey, respondents were asked for information about the number of studies underway at their sites. As was expected due to the overall number of studies underway across the country, the total number of studies in SMA varied quite a bit, while there was comparatively less variation within specific types of studies.

Table 4. Numbers of SMA Studies Underway at Sites (n=19)

	Minimum	Maximum	Mean
Phase I trials	0	2	0.7
Phase II trials	0	6	1.7
Phase III trials	0	4	1.3
Observational/natural history studies	0	3	1.1
Investigator-initiated studies	0	2	0.5
Open-label extension trials	0	3	1.2
Total # studies in SMA (sum)	1	11	5.3
Total # studies within unit (all diseases)*	2	40	14.3

*n=16; sites that reported >40 studies were excluded

To better see the relationship between the total number of studies and the research population, research population sizes were plotted against the total number of studies, as were the average number of SMA research patients per study at sites. In Phase III, this data will be analyzed on a site-specific level to better understand potential capacity across sites, as well as what sites' performance in terms of recruitment. This additional analysis will inform which sites are targeted as part of Phase III activities to increase capacity at sites.

Figure 4. Research Population in Relation to SMA Studies (n=19)

A. B.

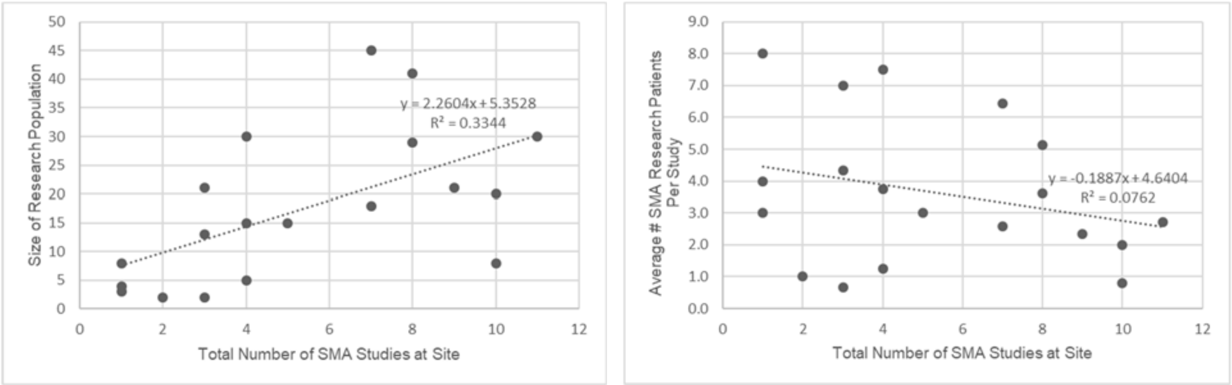

Figure A depicts the SMA research population vs, the total number of SMA studies. Figure B depicts the average number of SMA research patients vs the total number of studies at the site.

III. Site Staff

Respondents were asked for information about three categories of key staff (principal investigators, clinical trial coordinators, and physical therapists). The detailed results are summarized in the first table below. On average, sites had 4.2 total principal investigators in their unit. The average number of PIs with SMA experience across sites was 2.6. For clinical trial coordinators, sites had on average 6.3 clinical trial coordinators overall; the average number with SMA experience was 3.5. For physical therapists (PTs), sites had on average 5.7 physical therapists overall; the average number with SMA experience was 2.7. Across sites, PIs have on average have more experience with SMA (14.8 years) than either coordinators (3.5 years) or physical therapists (2.3 years).

Table 5. Principal Investigators, Clinical Trial Coordinators, and Physical Therapists: Total Numbers and Experience with SMA

	n	Min	Max	Mean	St Dev
Principal Investigators					
Total # of Principal Investigators*	18	1	10	4.2	2.62
# PIs with SMA experience	19	1	8	2.6	1.71
Average total years of experience	19	0	40	14.8	9.08
Average years experience in SMA	19	2	20	9.0	5.10
Clinical Trial Coordinators					
Total number of coordinators*	18	2	17	6.3	3.51
# coordinators with SMA experience	19	1	7	3.5	1.71
Average total years of experience	19	2	20	10.3	6.44
Average years experience in SMA	19	2	15	3.8	3.37
Physical Therapists					
Total number of PTs	19	0	35	5.7	7.94
# PTs with experience conducting SMA outcome measures in a clinical setting	19	2	5	2.7	1.00
Average years experience conducting SMA outcome measures in clinical trials	19	2	15	5.8	4.24
# PTs with reliability training for conducting SMA outcome measures in SMA clinical trials	19	1	5	2.3	0.82

*Excluded one site which reported 500 PIs and 150 coordinators.

Sites were also asked to report the numbers of other health care professionals they had on staff. Professionals were asked about staff important for providing comprehensive care to SMA patients such as neurologists, nurse practitioners, pulmonologists, GI specialists, occupational

and physical therapists. The second table in this section provides the summary of these results. The average numbers of these health care professionals at sites ranged from two to seven professionals, but there was large variability in staffing across sites. Individual sites had many of the additional support staff, but not necessarily all. No clear trends were identified when comparing the numbers of the key staff to the sizes of research population at SMA sites or the number of SMA studies underway. Sites were also asked to report the fraction of FTE time of these professionals that was dedicated to clinical trials, but an error in the survey prevented sites from providing meaningful information on this question (and a number of sites also reported that they do not calculate FTEs in this way). As a result, that information is not included here.

Table 6. Total Numbers of FTEs within Neurology Units (n=19)

	Minimum	Maximum	Mean	St Dev
Neurologists (n=17)*	2	8	4.1	2.00
Nurse practitioners (n=18)*	0	20	1.9	4.59
Pulmonologists	0	20	3.7	4.77
GI specialist/nutritionist/dieticians	0	23	3.1	5.17
Physical therapists	0	35	5.7	7.94
Occupational therapists	0	31	4.3	7.59
Electrophysiologists	0	7	2.2	1.77
Genetic counselors	0	10	1.9	2.90
Social workers	0	5	1.5	1.26
Principal Investigators	1	30	6.1	7.59
Clinical Research Coordinators	2	20	7.1	5.04

*Excluded two sites which reported >20 neurologists and one site which reported 200 nurse practitioners, as these numbers were dramatically higher than all other sites' numbers.

To understand the average patient load across sites, the minimum, maximum, and average ratios of research patient to staff across the sites were calculated. These data are summarized below. On average, sites have one PI for every 10 SMA research patients, one clinical research coordinator for every five research patients, and one physical therapists for every seven SMA research patients. Overall, there is one physical therapists for every 30 SMA patients in the total patient population for these sites.

Table 7. Key Health Care Professionals with SMA Experience: Total Numbers and Patient/ HCP Ratios (n=19)

	Minimum	Maximum	Median	Mean	St Dev
Numbers of PIs, Coordinators, and PTs with SMA Experience					
Principal Investigators	1	8	2	2.6	1.7
Clinical Research Coordinators	1	7	3	3.5	1.7
Physical Therapists	2	5	2.0	2.7	1.0
Ratios of Patients to Key Professionals with SMA Experience					
Research Patients/PIs	0.3	30.0	6.7	9.6	9.2
Research Patients/CRCs	0.5	10.0	5.0	5.1	3.0
Research Patients/PTs	1.0	15.0	6.5	6.8	4.9
Total Patients/PTs	6.3	71.7	27.3	30.2	17.9

IV. Site Capacity

Factors that Have Enabled Sites to Successfully Run Trials: Sites were asked to rank the significance of enabling factors including clinical staff bandwidth, staff and site coordination, performance of the clinical trials coordinator, staff expertise in SMA, and site infrastructure. No one factor clearly stood out as most important to successfully

running trials. Staff bandwidth, coordination, and performance of coordinators were all endorsed as extremely important to successful trial management.

Figure 5. Factors Enabling Sites to Successfully Run Trials (n=19)

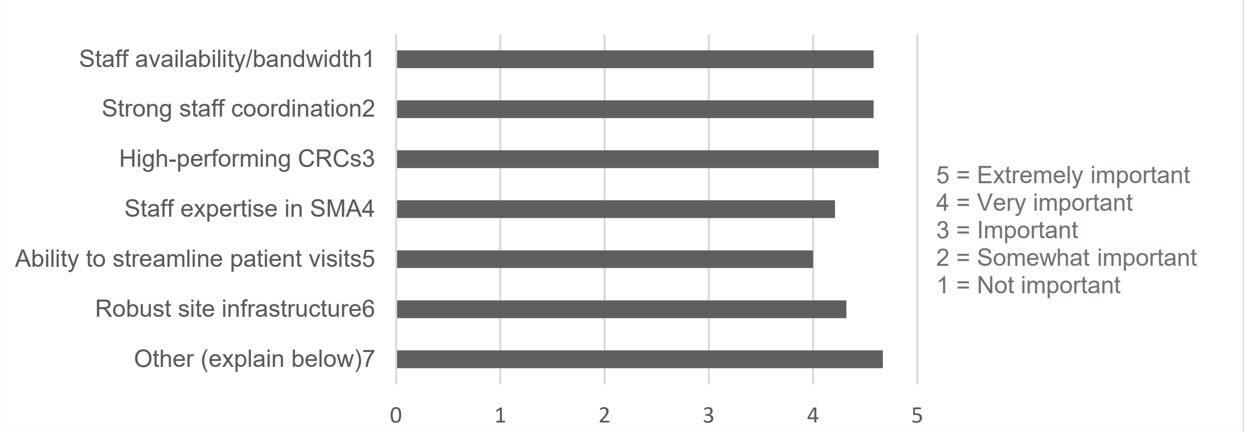

Participants were asked "How important have the following factors been in enabling you to successfully run clinical trials in SMA?"

1. Staff availability/bandwidth in general (WA=4.58)
2. Strong staff coordination in general (WA=4.58)
3. High-performing clinical trials coordinator (WA=4.63)
4. Staff expertise in SMA (WA=4.21)
5. Ability to streamline patient visits (e.g. by coordinating appointments to occur in a single day) (WA=4)
6. Robust site infrastructure (e.g. facilities well-suited to SMA patients/trials) (WA=4.32)
7. Other (explain below) (WA=4.32)

Factors Preventing Sites from Taking on Additional Trials: Sites were asked to rank the significance (on a Likert scale) of two categories of limiting factors related to (a) the site and its staff and (b) patients.

(a) Site and Staff Factors**:** Potential site and staff limiting factors covered by the survey were clinical staff bandwidth, trial staff bandwidth (including bandwidth of staff with SMA experience specifically), infrastructure, and other factors. Bandwidth of clinical and trial staff emerged as most the most significant factors limiting capacity across sites. However, these were only highly significant for a fraction of sites, and were not significant for about half of the sites. Bandwidth of staff with SMA expertise and limited site infrastructure did not appear to be significant factors. The graph below summarizes the weighted rankings for the individual factors.

Figure 6. Site/Staff-Related Factors Preventing Sites from Taking on Additional SMA Trials (n=19)

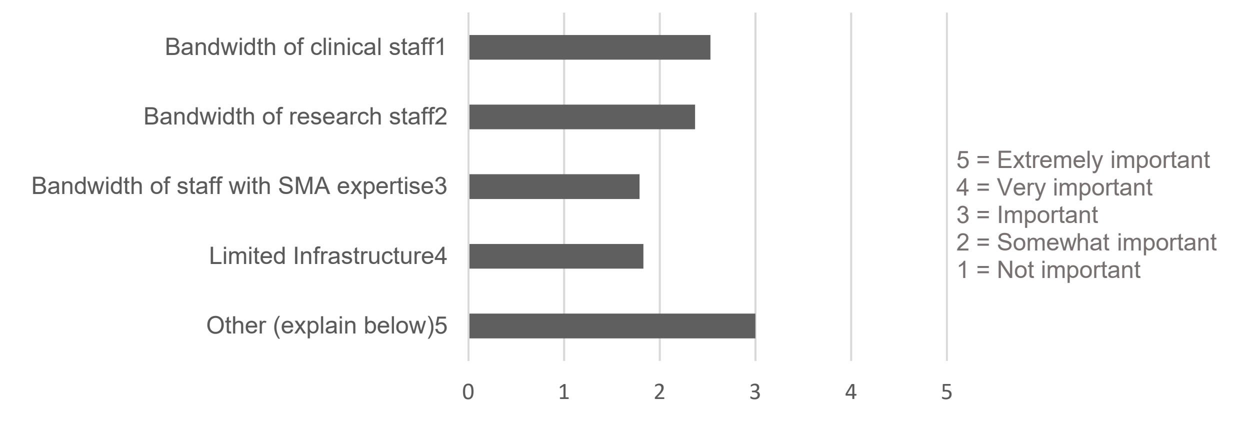

Participants were asked "how significant – if at all – are the following in preventing you from taking on additional SMA trials at this time?"

1. Bandwidth of clinical staff in general (WA=2.53)
2. Bandwidth of clinical trial (research) staff specifically (WA=2.37)
3. Bandwidth of clinical trials staff with SMA expertise (WA=1.79)
4. Limited Infrastructure (e.g. size or number of facilities) (WA=1.83)
5. Other (explain below) (WA=3)

(b) <u>Patient-Related Factors</u>***:*** Limiting factors related to patients that sites were asked about included patient interest, distance from sites, patient saturation due to ongoing open label extension trials, and increased numbers of patients being followed as new treatment options become available. All factors were endorsed as contributing, but there were not any individual factors that emerged as highly significant in limiting capacity. The graph below summarizes the weighted rankings for the individual factors.

Figure 7. Patient-Related Factors Preventing Sites from Taking on Additional SMA Trials (n=19)

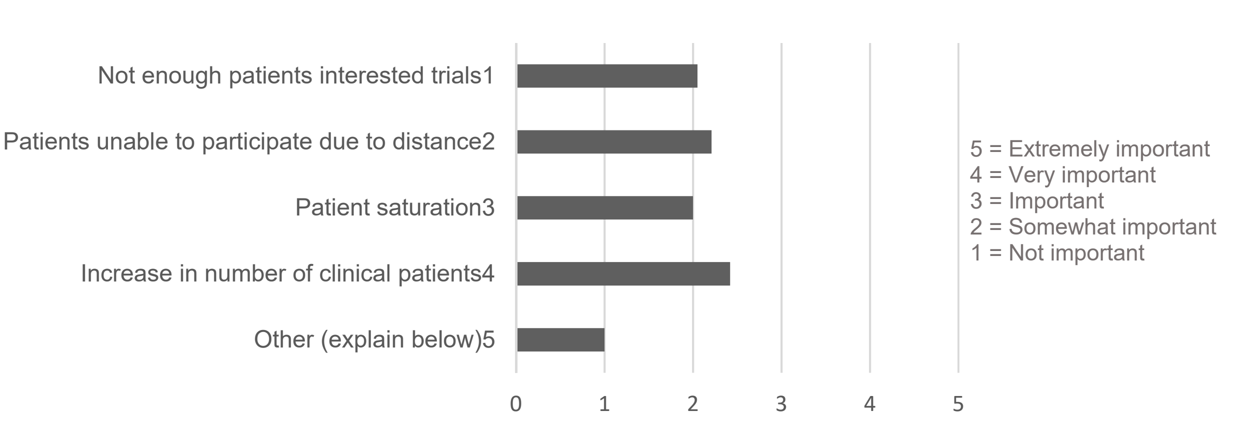

Participants were asked "how significant are the following in preventing you from taking on additional SMA trials at this time?"

1. Not enough patients interested in new trials (WA=2.05)
2. Patients interested in clinical trials but unable to participate due to distance from site (WA=2.21)
3. Patient saturation given existing number of patients being followed as part of open label extension trials (WA=2)
4. Increase in number of clinical patients being followed as new treatment options become available (WA=2.42)
5. Other (explain below) (WA=1)

Opportunities to increase capacity: Sites were asked to rank a series of potential approaches to addressing capacity challenges, and to provide free text suggestions for activities Cure SMA could undertake to support sites. Items ranked by sites included increasing numbers of clinical staff, trial staff, coordinators, principal investigators, and physical therapists; PT training in outcome measures; site visits with an experienced trial coordinator to identify opportunities to improve efficiency; and increased patient interest in trials.

Of these factors, increasing numbers of clinical staff, trial staff, and coordinators would be most helpful to sites. PT training in outcome measures and visits from coordinators would be helpful to a handful of sites. However, about half of the respondents indicated that these activities would not be helpful for their sites. The graph below summarizes the weighted rankings for the individual factors.

Figure 8. Factors Enabling Sites to Take on Additional SMA Trials (n=19)

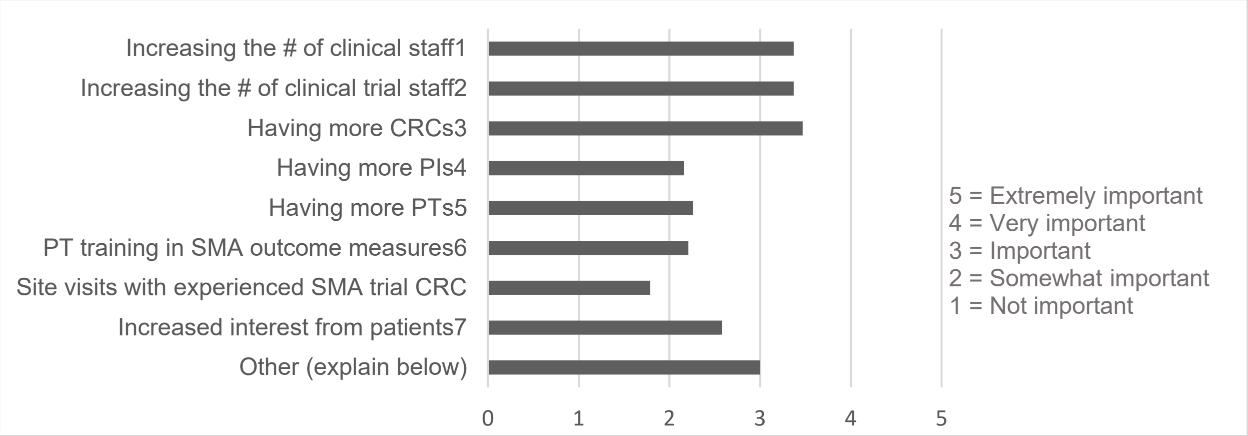

Participants were asked "how helpful would the following be in increasing your ability to take on additional SMA trials?"

1. Increasing the number of clinical staff in general (WA=3.37)
2. Increasing the number of clinical trial staff, specifically (WA=3.37)
3. Having more clinical research coordinators (WA=3.47)
4. Having more PIs (WA=2.16)
5. Having more PTs (WA=2.26)
6. PT training in SMA outcome measures (WA=2.21)
7. Site visits with experienced SMA trial coordinator to identify opportunities to improve efficiency (WA=1.79)
8. Increased interest from patients in clinical trials (WA=2.58)
9. Other (explain below) (WA=3)

Free-Text Responses: Sites were also given the opportunity to provide free text responses describing other activities Cure SMA might be able to do to help with management of existing patients and to help sites take on new clinical trials. Three themes were identified in the responses:

- Raising awareness of trials amongst the patient community in general was suggested by several respondents.
- Raising awareness about under-utilized sites and assistance with enrollment was also suggested by a handful of sites.
- Grants to support expanded infrastructure and coordinators were also identified as helpful by multiple sites.

Survey to Assess Clinical Trial Readiness and Capacity at SMA Clinical Trial Sites

Purpose

To better meet the needs of trial sponsors and the SMA patient community as the number of SMA clinical trials in the United States increases, Cure SMA and the Cure SMA Industry Collaboration (see below) are collectively interested in identifying and pursuing opportunities to increase trial site capacity and optimize readiness nationally. To this end, we are surveying clinical sites with experience conducting clinical trials in SMA to (a) understand sites' capacity for future clinical trials in SMA, (b) identify factors that may limit site trial capacity and/or readiness, and (c) identify opportunities to increase capacity.

The information collected through this survey will be used to develop a plan for alleviating challenges related to patient access to trial sites as well as site capacity, for the benefit of patients and the research community.

Target Audience

The target audience for this survey is clinical sites within the United States that have conducted clinical trials in SMA (Phase I-III). While the approach to completion of the survey is at the discretion of the site, it would be ideal - given the information requested - if a member of your research team involved in the day-to-day research activities at your site (e.g., a clinical trial coordinator) completes this survey.

Information Collected

This survey asks for general information about your clinical site along with its SMA patient population, capacity, clinical staff and clinical trial staff and their training, and operations. As described above, this information will be used by Cure SMA to develop a plan to help alleviate challenges related to patient access to trial sites as well as site capacity, for the benefit of patients, your site, and the research community.

Survey responses are being collected by Drinker Biddle & Reath LLP on behalf of Cure SMA and will be shared with Cure SMA. No identifiable responses will be shared beyond Cure SMA and Drinker Biddle & Reath LLP. De-identified responses will be shared with the SMA Industry Collaboration. If you have any questions about how your responses will be used, please contact Drinker Biddle & Reath LLP.

Important Note: Although one or more staff at your site may receive a unique link to this survey, we are requesting that only one survey be submitted, per site. Links may not be forwarded or otherwise shared, therefore we encourage you to use the PDF previously sent via email by Rosangel Cruz (Cure SMA) to facilitate internal data collection and coordinate a single response.

Survey Deadline

The response deadline for this survey is Friday, September 22, 2017.

Questions

We encourage you to contact us with any questions. You may email Rosangel Cruz at rosangel@curesma.org or Ilse Peterson (Drinker Biddle & Reath, LLP) at ilse.peterson@dbr.com.

Support for Survey

This effort is being supported through funds provided to Cure SMA in the context of the Cure SMA Industry Collaboration.

Cure SMA is a charitable organization dedicated to the treatment and cure of spinal muscular atrophy (SMA). Cure SMA provides support and information to families with children affected by SMA, and facilitates basic and clinical research aimed at preventing and treating SMA.

The Cure SMA Industry Collaboration is a collaboration of pharmaceutical and biotech companies, including Astellas, AveXis, Biogen, Genentech/Roche, Cytokinetics, and Ionis, involved in the development of SMA therapeutics. The objectives of the Cure SMA Industry Collaboration include leveraging the experience, expertise, and resources of pharmaceutical and biotech companies to advance best practices, standards and

approaches for development and clinical evaluation of therapeutics; enabling collaborative research; enhancing opportunities to engage health authorities in a patient-focused manner on topics related to drug development and review; sharing pertinent findings for the benefit of the broader scientific and regulatory community and the general public; and reducing patient fatigue through more streamlined and coordinated engagement of the patient and caregiver community.

Survey to Assess Clinical Trial Readiness and Capacity at SMA Clinical Trial Sites

GENERAL INFORMATION ABOUT YOUR SITE

* 1. Please provide the information below.

**Site Name**	
**Name of Respondent (First and Last Name)**	
**Role of Respondent within Organization**	
**Site location (City)**	
**Site location (State)**	-- select state --
**Email Address**	
**Phone Number**	

- Your survey answers are saved each time you click "**Next**".
- You may go back to change a response at any time before you submit your survey. If you exit the survey to resume later, you ***must*** use the link received in your emailed survey invitation.

Survey to Assess Clinical Trial Readiness and Capacity at SMA Clinical Trial Sites

INFORMATION ABOUT YOUR SITE'S SMA PATIENT POPULATION

This section asks for general information about the number, age, and source of referrals for SMA patients seen at your site.

* 2. How many SMA patients (all types) received routine care at your clinic/site within the last year?

\# of patients

* 3. How many SMA patients (all types) were seen at your site for clinical trials within the last year?

\# of patients

* 4. Of the SMA patients seen at your site for clinical trials, how many also received routine care and how many were seen only for research?

\# of patients seen for trials and routine care

\# of patients seen only for research

* 5. Do you see both children and adults?

- Children
- Adults
- Both

* 6. What is the oldest age of SMA patients you will see?

Age (years)

* 7. What is the youngest age (years) of SMA patients you will see? For less than 1 year, type '0'.

Age (years)

* 8. How important have the following factors been in driving referrals of SMA patients to your site?

	Not important	Somewhat important	Important	Very important	Extremely important
Lack of specialty care at referring site	◯	◯	◯	◯	◯
SMA expertise at your site	◯	◯	◯	◯	◯
Capacity at referring site	◯	◯	◯	◯	◯
Clinical trial(s) recruiting at your site	◯	◯	◯	◯	◯
Check here if reason for referral is unknown	◯	◯	◯	◯	◯
Other (explain below)	◯	◯	◯	◯	◯

Other (please explain)

- Your survey answers are saved each time you click " **Next**".
- You may go back to change a response at any time before you submit your survey. If you exit the survey to resume later, you ***must*** use the link received in your emailed survey invitation.

cure SMA Make today a breakthrough.

Survey to Assess Clinical Trial Readiness and Capacity at SMA Clinical Trial Sites

STUDIES UNDERWAY AT YOUR SITE

This section asks for general information about the number of clinical trials and other studies underway at your site.

* 9. Do you have a dedicated clinical research unit or clinical trials office?

- Yes
- No

* 10. How many clinical trials do you currently have underway, both overall, and in SMA specifically?

# of trials (total)

# of SMA trials

* 11. For SMA studies only, how many, if any, of the following types of studies are currently being conducted at your site? If none, please enter zero (0).

Phase I trials

Phase II trials

Phase III trials

Observational / Natural history studies

Investigator-initiated studies

Open label extension trials

- Your survey answers are saved each time you click "**Next**".
- You may go back to change a response at any time before you submit your survey. If you exit the survey to resume later, you ***must*** use the link received in your emailed survey invitation.

Survey to Assess Clinical Trial Readiness and Capacity at SMA Clinical Trial Sites

INFORMATION ABOUT SITE STAFF

This next section asks for information about research and clinical staff at your site, and their experience with clinical trials and outcome measures in SMA.

Principal Investigators

* 12. How many principal investigators (PIs) with experience conducting clinical trials (in any disease) do you have on staff?

# of PIs (total) [ ]

* 13. Of the PIs you have on staff, how many have experience conducting clinical trials specifically in SMA?

# of PIs with SMA-specific trial experience [ ]

* 14. On average, how many years of experience do PIs at your site have conducting clinical trials, both in general and in SMA specifically?

# years of trial experience overall [ ]

years of experience with SMA trials [ ]

Clinical Research Coordinators/Study Coordinators

* 15. How many clinical research coordinators do you have on staff?

# coordinators (total) [ ]

* 16. Of the coordinators that you have on staff, how many have experience conducting clinical trials specifically in SMA?

# coordinators with SMA-specific trial experience [ ]

* 17. On average, how many years of experience do the clinical research coordinators at your site have conducting clinical trials, both in general and in SMA specifically?

# years of trial experience overall

years of SMA-specific trial experience

Physical Therapists

* 18. How many physical therapists with experience conducting SMA-specific outcome measures in a clinical setting do you have on staff?

* 19. On average, how many years of experience do these physical therapists have conducting SMA-specific outcome measures **in SMA clinical trials**?

* 20. How many of your physical therapists have had reliability training for conducting SMA-specific outcome measures for SMA clinical trials?

Clinical Staff

* 21. How many of each of these types of professionals do you have on staff (in FTEs)?

Neurologists	
Nurse Practitioners (NPs)	
Pulmonologists	
GI specialist/Nutritionist/ Dieticians	
Physical Therapists	
Occupational Therapists (OTs)	
Electrophysiologists	
Genetic Counselors	
Social Workers	
Principal Investigators	
Clinical Research Coordinators	

* 22. For each type of professional, how many FTEs are dedicated to conducting clinical trials?

Neurologists	
Nurse Practitioners (NPs)	
Pulmonologists	
GI specialist/Nutritionist/ Dieticians	
Physical Therapists	
Occupational Therapists (OTs)	
Electrophysiologists	
Genetic Counselors	
Social Workers	

- Your survey answers are saved each time you click " **Next**".
- You may go back to change a response at any time before you submit your survey. If you exit the survey to resume later, you ***must*** use the link received in your emailed survey invitation.

SITE CAPACITY FOR CLINICAL TRIALS

This final section asks for information about factors that have enabled you to successfully run trials, factors that have prevented you from taking on additional trials, and what might help position you to take on additional trials.

* 23. How important have the following factors been in enabling you to successfully run clinical trials in SMA?

	Not important	Somewhat important	Important	Very important	Extremely important
Staff availability/bandwidth in general	○	○	○	○	○
Strong staff coordination in general	○	○	○	○	○
High-performing clinical trials coordinator	○	○	○	○	○
Staff expertise in SMA	○	○	○	○	○
Ability to streamline patient visits (e.g. by coordinating appointments to occur in a single day)	○	○	○	○	○
Robust site infrastructure (e.g. facilities well-suited to SMA patients/trials)	○	○	○	○	○
Other (explain below)	○	○	○	○	○

Other (please explain)

* 24. Does your site have the capacity to conduct additional SMA trials at this time?

○ Yes

○ No

* 25. Factors Related to Site and Staff

How significant – if at all – are the following in preventing you from taking on additional SMA trials at this time?

	Not significant	Somewhat significant	Significant	Very significant	Extremely significant
Bandwidth of clinical staff in general	○	○	○	○	○
Bandwidth of clinical trial (research) staff specifically	○	○	○	○	○
Bandwidth of clinical trials staff with SMA expertise	○	○	○	○	○
Limited Infrastructure (e.g. size or number of facilities)	○	○	○	○	○
Other (explain below)	○	○	○	○	○

Other (please explain)

* 26. Factors Related to Patient Population

How significant are the following in preventing you from taking on additional SMA trials at this time?

	Not significant	Somewhat significant	Significant	Very significant	Extremely significant
Not enough patients interested in new trials	○	○	○	○	○
Patients interested in clinical trials but unable to participate due to distance from site	○	○	○	○	○
Patient saturation given existing number of patients being followed as part of open label extension trials	○	○	○	○	○
Increase in number of clinical patients being followed as new treatment options become available	○	○	○	○	○
Other (explain below)	○	○	○	○	○

Other (please explain)

* 27. How helpful would the following be in increasing your ability to take on additional SMA trials?

	Not helpful	Somewhat helpful	Helpful	Very helpful	Extremely helpful
Increasing the number of clinical staff in general	○	○	○	○	○
Increasing the number of clinical trial staff, specifically	○	○	○	○	○
Having more clinical research coordinators	○	○	○	○	○
Having more PIs	○	○	○	○	○
Having more PTs	○	○	○	○	○
PT training in SMA outcome measures	○	○	○	○	○
Site visits with experienced SMA trial coordinator to identify opportunities to improve efficiency	○	○	○	○	○
Increased interest from patients in clinical trials	○	○	○	○	○
Other (explain below)	○	○	○	○	○

Other (please explain)

* 28. Is there anything Cure SMA might be able to do to help with the management of existing patients and to help you take on new clinical trials at your site?

- Your survey answers are saved each time you click "**Next**".
- You may go back to change a response at any time before you submit your survey. If you exit the survey to resume later, you ***must*** use the link received in your emailed survey invitation.

Survey to Assess Clinical Trial Readiness and Capacity at SMA Clinical Trial Sites

FOLLOW-UP

29. May we reach out to you with follow-up questions (e.g., to request clarifying information related to responses above)?

- ◯ Yes
- ◯ No

- You have reached the end of the survey.
- You may go back to change a response or exit the survey and resume later, however you ***must*** use the link received in your emailed survey invitation.
- After you click "Submit survey", your responses are locked, finalized and submitted.
