# Supplementary material for: The SMA Clinical Trial Readiness Program: creation and evaluation of a program to enhance SMA trial readiness in the United States
Source: Orphanet J Rare Dis. 2020 May 22;15:118. doi: 10.1186/s13023-020-01387-8 (PMC7564894; doi:10.1186/s13023-020-01387-8)
Supplement: Supplementary file 3 — Additional file 3. Site evaluation questionnaires used in 2018. [file 13023_2020_1387_MOESM3_ESM.pdf]

Cure SMA Clinical Trial Readiness Pre-Screen

Pre-Screen Background and Instructions

Thank you for your interest in Cure SMA's readiness evaluation. This evaluation is part of ongoing efforts by Cure SMA and the SMA Industry Collaboration (IC)* to increase trial site capacity and optimize readiness throughout the United States, to better meet the needs of trial sponsors and the SMA patient community as the number of SMA clinical trials increases.

Overview of Evaluation Process

The readiness evaluation consists of a two-step process designed to assess sites' readiness to conduct clinical trials in SMA. It includes:

1. **A 10-question pre-screen (this survey) that asks for basic information related to clinical trial experience and conduct, which should be completed by a principal investigator at your site (or someone else with knowledge of your site's staff, resources, and SMA patient population).**
2. **An in-depth evaluation that includes completion of a full trial readiness checklist and a phone call with our team to review your completed checklist and discuss any potential gaps to conducting effective trials in SMA.**

This survey constitutes the pre-screen, and may be completed and submitted anytime. Once you submit your pre-screen, you can expect to hear from us within ten business days about next steps. If you meet the pre-screen requirements -- which simply include (1) that your site currently sees SMA patients and (2) that you have clinical trial experience and infrastructure -- we will send you the in-depth evaluation for completion.

After You Have Completed the Evaluation

If you complete the full readiness evaluation process, we will coordinate to make – with your permission – your completed readiness checklist available to clinical trial sponsors who may be seeking new trial sites. We will also recommend resources that may help you to enhance your site's readiness over time, and address any gaps identified during the evaluation process**

We appreciate your interest in helping to meet the needs of SMA patients and trial sponsors by participating in this process.

The Cure SMA Industry Collaboration is a collaboration of pharmaceutical and biotech companies, including Astellas, AveXis, Biogen, Genentech/Roche, and Cytokinetics, involved in the development of SMA therapeutics. The objectives of

the Cure SMA Industry Collaboration include leveraging the experience, expertise, and resources of pharmaceutical and biotech companies to advance best practices, standards and approaches for development and clinical evaluation of therapeutics; enabling collaborative research; enhancing opportunities to engage health authorities in a patient-focused manner on topics related to drug development and review; sharing pertinent findings for the benefit of the broader scientific and regulatory community and the general public; and reducing patient fatigue through more streamlined and coordinated engagement of the patient and caregiver community.

*****When you submit your in-depth evaluation in step two, we will ask for permission to share your completed evaluation with trial sponsors. Sharing this information with sponsors is not required for participation in this initiative, and you will be able to opt-out if desired. Further, participation in this process will not guarantee that you will be contacted by a sponsor or asked to participate in a trial. It will ultimately be the decision of trial sponsors to determine which sites they contact, and when.***

Cure SMA Clinical Trial Readiness Pre-Screen

General Information

1. Please provide the following information:

Investigator Name

Institution Name

Department

Location (City, State)

Phone Number

2. Does your site have clinical research infrastructure?

- Yes
- No

3. Is your site currently seeing SMA patients for research or care?

- Yes
- No

Cure SMA Clinical Trial Readiness Pre-Screen

Part 1 of 2: Experience with Clinical Trials

4. Please provide the following information about your site's experience with clinical trials:

	Yes	No
Is your site currently conducting clinical trials?	◯	◯
Has your site conducted clinical trials in the past?	◯	◯
Is your site currently conducting NEUROMUSCULAR trials?	◯	◯
Has your site conducted NEUROMUSCULAR trials in the past?	◯	◯

5. Does your site have at least one principal investigator who is a neurologist and has experience conducting clinical trials in the following areas? Check all that apply.

- ☐ Clinical trials in SMA
- ☐ Clinical trials in other neuromuscular diseases (but not SMA)
- ☐ Clinical trials in any other disease area (specify below)

Please specify, if applicable, in which other disease area(s) the principle investigator has trial experience:

6. Does your site have at least one clinical research coordinator with experience conducting clinical trials in the following areas? Check all that apply.

- ☐ Clinical trials in SMA
- ☐ Clinical trials in other neuromuscular diseases (but not SMA)
- ☐ Clinical trials in any other disease area

7. Does your site have at least one physical therapist with experience conducting the following outcome measures? Check all that apply.

- ☐ SMA-specific motor function outcome measures, clinical evaluation only
- ☐ SMA-specific motor function outcome measures in clinical trials
- ☐ Outcome measures for other neuromuscular diseases (but not SMA), clinical evaluation only
- ☐ Outcome measures for other neuromuscular diseases (but not SMA) in clinical trials

Cure SMA Clinical Trial Readiness Pre-Screen

Part 2 of 2: Questions Regarding Clinical Trial Operations

8. Do you work with a local or a centralized IRB?

- ○ Yes
- ○ No

Please specify which IRB:

____

9. Do you have an established process for ensuring adherence to standard of care for SMA?

- ○ Yes
- ○ No

10. Do you have an established and well-documented process for ensuring adherence to study protocols?

- ○ Yes
- ○ No

Cure SMA Clinical Trial Readiness Pre-Screen

Thank You & Next Steps

We appreciate your interest in helping to meet the needs of SMA patients and trial sponsors by participating in this process. You can expect to hear from us within 10 business days about next steps. If your site meets the pre-screen requirements, we will send you the in-depth evaluation for completion.

Site Readiness Checklist for Clinical Trials
Step 2 of Readiness Evaluation Process

Background and Instructions

Thank you for your interest in Cure SMA's clinical trial readiness evaluation. This evaluation is part of ongoing efforts by Cure SMA and the SMA Industry Collaboration (IC)* to increase trial site capacity and optimize readiness throughout the United States, to better meet the needs of trial sponsors and the SMA patient community as the number of SMA clinical trials increases.

Overview of Readiness Evaluation

The readiness evaluation consists of a two-step process designed to assess sites' readiness to conduct clinical trials in SMA. It includes:

1. **A 10-question pre-screen that asks for basic information related to clinical trial experience and conduct, which should be completed by a principal investigator at your site (or someone else with knowledge of your site's staff, resources, and SMA patient population).**
2. **An in-depth evaluation that includes completion of a full trial readiness checklist (this checklist) and a phone call with our team to review your completed checklist and discuss potential gaps in readiness for conducting trials in SMA, as well as resources that may help you to enhance your site's readiness.**

This checklist is part of the in-depth evaluation, and may be completed and submitted to the Cure SMA Clinical Trial Readiness Team (rosangel@curesma.org and ilse.peterson@dbr.com) anytime (please type responses to each question here). Once we receive your completed checklist, we will reach out within 10 business days to schedule a phone interview.

After You Have Completed the Evaluation

After you complete the evaluation process, we will make information about your participation in this process available to clinical trial sponsors, including Cure SMA Industry Collaboration members, who may be seeking new trial sites.* At that time, you may also elect to have Cure SMA share your completed readiness checklist with these sponsors.

We appreciate your interest in helping to meet the needs of SMA patients and trial sponsors.

By submitting this checklist, you are agreeing that Cure SMA may disclose that you have participated in this process to members of the Cure SMA Industry Collaboration. The Cure SMA Industry Collaboration is a collaboration of pharmaceutical and biotech companies, including Astellas, AveXis, Biogen, Genentech/Roche, and Cytokinetics, involved in the development of SMA therapeutics. The objectives of the Cure SMA Industry Collaboration include leveraging the experience, expertise, and resources of pharmaceutical and biotech companies to advance best practices, standards and approaches for

development and clinical evaluation of therapeutics; enabling collaborative research; enhancing opportunities to engage health authorities in a patient-focused manner on topics related to drug development and review; sharing pertinent findings for the benefit of the broader scientific and regulatory community and the general public; and reducing patient fatigue through more streamlined and coordinated engagement of the patient and caregiver community.

SECTION 1. GENERAL INFORMATION

Please provide the information below.

Principal Investigator Name

Institution Name

Department

Location (City, State)

Date Submitted to Cure SMA

- Your survey answers are saved each time you click "**Next**".
- You may go back to change a response at any time before you submit your survey. If you exit the survey to resume later, you ***must*** use the link received in your emailed survey invitation.

Site Readiness Checklist for Clinical Trials
Step 2 of Readiness Evaluation Process

SECTION 2. STUDIES UNDERWAY AT SITE

2.1 Do you have a dedicated clinical research unit?

- ○ Yes
- ○ No

2.2 Are you currently conducting clinical trials at your site?

- ○ Yes
- ○ No, but I have in the past
- ○ No, and I have not in the past

- Your survey answers are saved each time you click "**Next**".
- You may go back to change a response at any time before you submit your survey. If you exit the survey to resume later, you ***must*** use the link received in your emailed survey invitation.

SECTION 2. STUDIES UNDERWAY AT SITE

Since you are currently conducting clinical trials at your site, please indicate your experience conducting clinical trials with the following populations:

- ◯ Pediatric only
- ◯ Both pediatric and adult
- ◯ Adult only

- Your survey answers are saved each time you click "**Next**".
- You may go back to change a response at any time before you submit your survey. If you exit the survey to resume later, you ***must*** use the link received in your emailed survey invitation.

SECTION 2. STUDIES UNDERWAY AT YOUR SITE

2.3 How many clinical research studies for neuromuscular diseases are currently active at your site? Please include investigator initiated, observational (e.g. natural history) and clinical trials (all phases). Count ongoing studies and studies that have been approved but may not be enrolling yet.

2.4 Are any of the research studies listed above (question 2.3) in SMA?

- Yes
- No

- Your survey answers are saved each time you click "**Next**".
- You may go back to change a response at any time before you submit your survey. If you exit the survey to resume later, you ***must*** use the link received in your emailed survey invitation.

SECTION 2. STUDIES UNDERWAY AT YOUR SITE

2.5a If you have active research studies in **SMA Type I**, please indicate the total number of active studies (SMA Type I only). **Please count each study only once. Do not count a single study under multiple categories.*

Investigator-Initiated

Observational (natural history)

Phase I

Phase I/II

Phase II

Phase III

Open Label Extension

2.5b If you have active research studies in **SMA Type II/III**, please indicate the total number of active studies (SMA Type II/III only). **Please count each study only once. Do not count a single study under multiple categories.*

Investigator-Initiated

Observational (natural history)

Phase I

Phase I/II

Phase II

Phase III

Open Label Extension

2.6 How many of the active SMA studies are currently enrolling?

2.7 How many patients have been enrolled in each neuromuscular study (including SMA studies) conducted at your site? Please list each study and the corresponding number of patients enrolled.

2.8 On how many clinical studies do you currently serve as PI?

Total Studies:

SMA Studies:

- Your survey answers are saved each time you click "**Next**".
- You may go back to change a response at any time before you submit your survey. If you exit the survey to resume later, you ***must*** use the link received in your emailed survey invitation.

SECTION 3. INFORMATION ABOUT SITE'S SMA PATIENT POPULATION

3.1 How many SMA patients were seen (for care or clinical trials) at your site within the last 12 months?

Type I:

Type II:

Type III:

3.2 What ages of patients will you see?

- ☐ Children ages
- ☐ Adults ages

Please specify the age range(s).

3.3 What percentage of SMA patients seen at your site (for care or clinical trials) were also diagnosed at your site?

- ○ None
- ○ 1 - 25%
- ○ 26 - 50%
- ○ 51 - 75%
- ○ 76 - 100%

3.4 What percentage of SMA patients, seen at your site, were referred to your site (for care or clinical trials) by another clinical site or by other organization, (e.g. patient advocacy organization)?

- ○ None
- ○ 1 - 25%
- ○ 26 - 50%
- ○ 51 - 75%
- ○ 76 - 100%

3.5 <u>Of the patients referred to your site, in the last year</u>, what percentage was referred specifically to participate in a research study?

- ◯ None
- ◯ 1 - 25%
- ◯ 26 - 50%
- ◯ 51 - 75%
- ◯ 76 - 100%

3.6 What percentage of SMA patients (seen or followed at your clinic) are located within 180 miles of your practice?

- ◯ None
- ◯ 1 - 25%
- ◯ 26 - 50%
- ◯ 51 - 75%
- ◯ 76 - 100%

- Your survey answers are saved each time you click "**Next**".
- You may go back to change a response at any time before you submit your survey. If you exit the survey to resume later, you ***must*** use the link received in your emailed survey invitation.

SECTION 4. INFORMATION ABOUT RESEARCH STAFF

Principal Investigator

4.1 How many years of experience do you have conducting clinical trials (in any area)?

4.2 How many years of experience do you have conducting research studies and clinical trials in SMA (if applicable)?

Years of experience conducting research studies in SMA:

Years of experience conducting SMA clinical trials specifically:

4.3 Have you completed the Association of Clinical Research Professionals' clinical research certification for PIs (Certified Principal Investigator)?

- Yes
- No

If yes, what year did you first receive certification?

4.4 Please list any additional investigator-related training/certification(s) in the conduct of clinical research.

(list training, names of individuals who have completed them, and dates)

Clinical Research Coordinators/Study Coordinators

4.5 How many clinical research coordinators do you have available on staff that could support SMA research studies?

4.6 How many years of clinical research experience does this (do these) coordinator(s) have?

(Please list the names of coordinators and years of experience with clinical research.)

4.7 For clinical research coordinators or study coordinators with SMA trial experience, what is the average number of years of experience conducting SMA trials? (Leave blank if none of the coordinators at your site have SMA experience.)

(Please list the names of coordinators and years of experience with clinical research in SMA)

4.8 How many studies at your site are actively enrolling and how many patients (on average) does each coordinator typically manage?

actively enrolling studies:

patients typically managed by each coordinator:

4.9 Have coordinators at your site completed the Association of Clinical Research Professionals' Clinical Research Coordinator certification?

(Please list the names of certified coordinators and years in which certifications were completed.)

4.10 Have coordinators at your site completed the Society of Clinical Research Associates' (SOCRA) clinical research certification? Please list the names of all coordinators who have completed certifications, and the years in which the certifications were completed.

(Please list the names of certified coordinators and years in which certifications were completed.)

4.11 Please list any additional CRC-related training/certification(s) in the conduct of clinical research (list names and dates), by coordinator.

(List other applicable trainings and dates, with the names of individuals who have completed them.)

Physical Therapists

4.12 How many physical therapists do you have on staff with experience conducting motor-function specific outcome measures in clinical trials (in any disease)?

4.13 How many of physical therapists do you have on staff with experience conducting SMA-specific functional-outcome measures in SMA clinical trials?

4.14 How many years of experience does each physical therapist have conducting SMA-specific functional-outcome measures?

(List PT names, which SMA population(s) they have experience with (i.e. Type I, Type II, Type III), and the number of years of experience with these populations)

4.15 How many physical therapists/evaluators are typically assigned per each study/trial?

(List # of evaluators assigned per each study/trial.)

4.16 How many PT evaluators at your site have completed reliability training for these motor function outcome measures?

Children's Hospital of Pennsylvania (CHOP):

Motor Function Measure (MFM):

Hammersmith Functional Motor Scale Expanded (HFMSE):

Upper Limb Module/Revised Upper Limb Module:

Bayley–III:

Six-Minute Walk Test:

Hand-held dynamometry:

Other (please list outcome measure and number of people who have completed reliability training):

Staff Training Pertaining to the Responsible and Ethical Conduct of Clinical Research

4.17 Have all relevant staff involved in clinical trials received the following types of training and certifications?

- [ ] Good Clinical Practices (GCP) Certification (All)
- [ ] CITI Training on Human Subjects Research
- [ ] Process of writing and administering informed consent (PI/CRC)
- [ ] Basics of Clinical Research (history of clinical trials (declaration of Helsinki)
- [ ] FDA Regulated Research (CITI Module or through an academic institution)
- [ ] Research With Minors (CITI Module/or through an academic institution)
- [ ] HIPAA compliance training
- [ ] Bloodborne Pathogens (CRC/as pertinent to role)
- [ ] IATA Dangerous Goods (for shipping of clinical samples) (CRC/as pertinent to role)
- [ ] Other (Please list any relevant not listed above):

- Your survey answers are saved each time you click "**Next**".
- You may go back to change a response at any time before you submit your survey. If you exit the survey to resume later, you ***must*** use the link received in your emailed survey invitation.

SECTION 5. INFORMATION ABOUT OTHER CLINICAL STAFF

5.1 How many of each of these types of professionals do you have on staff?

Neurologists: ___

Cardiologists: ___

Orthopedic Surgeons: ___

Nurse Practitioners (NPs): ___

Pulmonologists: ___

Respiratory Therapists: ___

GI specialist: ___

Nutritionist/Dieticians: ___

Physical Therapists: ___

Occupational Therapists: ___

Electrophysiologists: ___

Genetic Counselors: ___

Social Workers: ___

- Your survey answers are saved each time you click "**Next**".
- You may go back to change a response at any time before you submit your survey. If you exit the survey to resume later, you ***must*** use the link received in your emailed survey invitation.

SECTION 6. COORDINATION OF PATIENT CARE

6.1 Do you have a process for coordinating the care of research patients recruited at your site but whose medical home is outside your institution?

- ◯ Yes
- ◯ No

If yes, please briefly describe how matters pertaining to research and care follow up are communicated and coordinated) (that is, incidental findings at a research vist or updates on changes in health status)?

6.2 What resources does your clinic provide for patient care coordination?

- ◯ Dedicated Care Coordinator
- ◯ Other (if other, please describe below)

6.3 How do you ensure that your patients are receiving standard of care?

SECTION 7. ADDITIONAL QUESTIONS REGARDING CLINICAL TRIAL OPERATIONS

7.1 Do you have a centralized IRB? If not, please indicate the local IRB with which you intend to work.

- ◯ Yes
- ◯ No

Local IRB, if applicable:

7.2 Do you have an institutional biosafety committee?

- ◯ Yes
- ◯ No

7.3 Do you have a well-documented informed consent process?

- ◯ Yes
- ◯ No

7.4 Do you have an established and well-documented process for ensuring adherence to the study protocol?

- ◯ Yes
- ◯ No

If yes, please provide a high level overview of your process:

7.5 Do you have an established and well-documented approach to PI oversight, in particular for addressing serious adverse events and managing records?

- ◯ Yes
- ◯ No

7.6 Please describe your site's storage capabilities for investigational products and biological samples (e.g. freezers, sample chain of custody, access restrictions, etc.).

7.7 Does your clinical research unit conduct regular meetings? If so, how often?

- ○ Yes
- ○ No

Meeting Frequency:

7.8 Have you or your practice been inspected by the FDA or a similar regulatory agency in the past five years? If yes, what was the outcome?

- ○ Yes
- ○ No

Outcome:

7.9 Has your site ever been closed by a sponsor?

- ○ Yes
- ○ No

SECTION 7. ADDITIONAL QUESTIONS REGARDING CLINICAL TRIAL OPERATIONS

Why was your site closed by a sponsor?*(Please check all that aplly.)*

- ☐ Enrollment issues
- ☐ Conduct issues
- ☐ Other (please briefly describe)

SECTION 8. READINESS TO CONDUCT FUTURE RESEARCH & ADDITIONAL COMMENTS

8.1 Please describe any additional capabilities at your site or experience from other neuromuscular clinical trials that would support your ability to perform SMA research studies.

8.2 Are there any existing gaps/concerns that would prevent you from conducting new clinical trials in SMA immediately or in the near future?

- Yes
- No

If yes, please describe:

8.3 How could the Cure SMA readiness program best help you to meet your goal of becoming an SMA clinical trial site?

- You have reached the end of the survey.

- You may go back to change a response or exit the survey and resume later, however you ***must*** use the link received in your emailed survey invitation.

- After you click "Submit survey", your responses are locked, finalized and submitted.
