# Supplementary material for: The SMA Clinical Trial Readiness Program: creation and evaluation of a program to enhance SMA trial readiness in the United States
Source: Orphanet J Rare Dis. 2020 May 22;15:118. doi: 10.1186/s13023-020-01387-8 (PMC7564894; doi:10.1186/s13023-020-01387-8)
Supplement: Supplementary file 4 — Additional file 4. Streamlined site evaluation questionnaire used in 2019. [file 13023_2020_1387_MOESM4_ESM.pdf]

Site Readiness Questionnaire for SMA Clinical Trials (2019)

INTRODUCTION

Background and Instructions

Thank you for your interest in Cure SMA's clinical trial readiness evaluation. This evaluation is part of ongoing efforts by Cure SMA and the SMA Industry Collaboration (IC)* to increase trial site capacity and optimize readiness throughout the United States as the number of SMA clinical trials grows.

Overview of Readiness Evaluation

This process is designed to assess site readiness to conduct clinical trials in SMA and includes:

1. An online questionnaire about your site and team's experience with clinical trials and SMA patients, your SMA patient population, approaches to care coordination, and site infrastructure.
2. A phone interview to discuss your completed questionnaire, potential opportunities to increase readiness, and specific resources to enhance readiness.

A PDF copy of this survey is provided to facilitate internal coordination. Please use the PDF as a reference, but respond via the online survey. Once we receive your response, we will reach out within 10 business days to schedule a phone interview. If you have any questions, please contact our Cure SMA Clinical Trial Readiness Team, Rosangel Cruz (rosangel@curesma.org) and Ilse Peterson (ilse.peterson@dbr.com).

Eligibility Criteria

To be eligible to participate in this process, you and your site must:

1. See SMA patients
2. Have experience conducting clinical trials
3. Have familiarity with motor function outcome measures in SMA

After You Have Completed the Evaluation

After you have completed this evaluation, we will share information about your participation in

this process with clinical trial sponsors, including Cure SMA Industry Collaboration members who may be seeking new trial sites. At that time, you may also elect to have Cure SMA share your completed readiness checklist with these sponsors.

We appreciate your interest in helping to meet the needs of SMA patients and trial sponsors.

**The Cure SMA Industry Collaboration was established in 2016 to leverage the experience, expertise, and resources of pharmaceutical, biotechnology companies and other nonprofit organizations involved in the development of SMA therapeutics to more effectively address a range of scientific, clinical and regulatory challenges. Current members include Astellas, AveXis, Biogen, Cytokinetics, Genentech/Roche, Scholar Rock, and Novartis.*

SECTION 1. GENERAL INFORMATION

Page exit logic: Skip / Disqualify Logic
IF: Question "The eligibility criteria to participate in this evaluation process are as follows:

- Your site has clinical research infrastructure
- Your site is currently seeing SMA patients for research or care

Please confirm the following statements:" is not exactly equal to ("Our site has clinical research infrastructure","Our site is currently seeing SMA patients for research or care")
THEN: Jump to page 10 - End of Survey Flag response as complete

Please provide the information below. *

Principal Investigator (PI) Name

PI Specialty

PI Phone #

PI Email

Institution Name

Department

Location (City, State)

The eligibility criteria to participate in this evaluation process are as follows:

- Your site has clinical research infrastructure
- Your site is currently seeing SMA patients for research or care

Please confirm the following statements:

☐ Our site has clinical research infrastructure

☐ Our site is currently seeing SMA patients for research or care

To save and exit, click the "Save and Continue Later" option displayed in the gray bar at the bottom of the survey browser window. You will be prompted to enter your email address and will receive a unique link, which will allow you to complete your responses at a later time.

SECTION 2. STUDIES UNDERWAY AT SITE

2.1 Do you have a dedicated clinical research unit?

- ○ Yes
- ○ No

2.2 Please indicate your experience conducting clinical trials with the following populations:

- ○ Pediatric only
- ○ Adult only
- ○ Both pediatric and adult

2.3 Please provide the following information about your site's experience with **clinical trials**:

	Yes	No
Is your site currently conducting clinical trials?	○	○
Has your site conducted clinical trials in the past?	○	○
Is your site currently conducting neuromuscular trials?	○	○
Has your site conducted neuromuscular trials in the past?	○	○
Is your site currently conducting SMA trials?	○	○
Has your site conducted SMA trials in the past?	○	○

VALIDATION Must be numeric Whole numbers only

LOGIC Hidden unless: Question "Is your site currently conducting neuromuscular trials?" is one of the following answers ("Yes")

2.3.1 How many **NEUROMUSCULAR** clinical trials is your site currently conducting?

____

2.4 Are you currently conducting any research studies or clinical trials in SMA?

- ☐ Clinical trials
- ☐ Other research studies (not trials, e.g., investigator-initiated or natural history studies)

2.5 Please provide information on each SMA research study (including

clinical trials if applicable) underway at your site.

	Study Name	Study Type	SMA Type	Actively Enrolling		# Patients Enrolled
				Yes	No	
1		Investigator-Initiated<br>Observational (natural history)<br>Phase I<br>Phase I/II<br>Phase II<br>Phase III<br>Open Label Extension	Type I<br>Type II<br>Type III			
2		Investigator-Initiated<br>Observational (natural history)<br>Phase I<br>Phase I/II<br>Phase II<br>Phase III<br>Open Label Extension	Type I<br>Type II<br>Type III			
3		Investigator-Initiated<br>Observational (natural history)<br>Phase I<br>Phase I/II<br>Phase II<br>Phase III<br>Open Label Extension	Type I<br>Type II<br>Type III			
4		Investigator-Initiated<br>Observational (natural history)<br>Phase I<br>Phase I/II<br>Phase II<br>Phase III<br>Open Label Extension	Type I<br>Type II<br>Type III			
5		Investigator-Initiated<br>Observational (natural history)<br>Phase I<br>Phase I/II	Type I<br>Type II<br>Type III			

		Phase II<br>Phase III<br>Open Label Extension	Type III			
6		Investigator-Initiated<br>Observational (natural history)<br>Phase I<br>Phase I/II<br>Phase II<br>Phase III<br>Open Label Extension	Type I<br>Type II<br>Type III	○	○	
7		Investigator-Initiated<br>Observational (natural history)<br>Phase I<br>Phase I/II<br>Phase II<br>Phase III<br>Open Label Extension	Type I<br>Type II<br>Type III	○	○	
8		Investigator-Initiated<br>Observational (natural history)<br>Phase I<br>Phase I/II<br>Phase II<br>Phase III<br>Open Label Extension	Type I<br>Type II<br>Type III	○	○	
9		Investigator-Initiated<br>Observational (natural history)<br>Phase I<br>Phase I/II<br>Phase II<br>Phase III<br>Open Label Extension	Type I<br>Type II<br>Type III	○	○	
10		Investigator-Initiated<br>Observational (natural history)<br>Phase I<br>Phase I/II<br>Phase II<br>Phase III<br>[illegible]	Type I<br>Type II<br>Type III	○	○	

Open Label Extension

To save and exit, click the "Save and Continue Later" option displayed in the gray bar at the bottom of the survey browser window. You will be prompted to enter your email address and will receive a unique link, which will allow you to complete your responses at a later time.

SECTION 3. INFORMATION ABOUT SITE'S SMA PATIENT POPULATION

VALIDATION Must be numeric

3.1 How many SMA patients were seen at your site for care and/or clinical research within the last 12 months?

	# Seen for Only for Care	# Seen for Only for Research	# Seen for Care and Research
Type I:			
Type II:			
Type III:			

VALIDATION Must be numeric Whole numbers only

3.2 What ages of patients do you see?

	Youngest Age	Oldest Age
Children		
Adults		

3.3 What percentage of SMA patients seen at your site (for care or clinical research) were also diagnosed at your site?

- None
- 1 - 25%
- 26 - 50%
- 51 - 75%
- 76 - 100%

3.4 What percentage of SMA patients seen at your site were referred to your site (for care or clinical research) by another clinical site or by other organization (e.g. patient advocacy organization)?

- None
- 1 - 25%
- 26 - 50%
- 51 - 75%
- 76 - 100%

3.5 Of the patients referred to your site **in the last year,** what percentage were referred specifically to participate in a research study?

- None
- 1 - 25%
- 26 - 50%
- 51 - 75%
- 76 - 100%

3.6 What percentage of SMA patients (seen or followed at your clinic) live within 180 miles of your practice?

- None
- 1 - 25%
- 26 - 50%
- 51 - 75%
- 76 - 100%

To save and exit, click the "Save and Continue Later" option displayed in the gray bar at the bottom of the survey browser window. You will be prompted to enter your email address and will receive a unique link, which will allow you to complete your responses at a later time.

SECTION 4. INFORMATION ABOUT RESEARCH TEAM

PRINCIPAL INVESTIGATOR

4.1 On how many clinical studies do you currently serve as principal investigator?

Total Studies

SMA Studies

VALIDATION Must be numeric Whole numbers only

4.2 How many years of experience do you have conducting clinical trials and other clinical research studies (e.g. natural history and investigator-initiated studies)?

Clinical trials (any area)

Neuromuscular clinical trials

SMA clinical trials

Neuromuscular research studies (not trials)

SMA research studies (not trials)

4.3 What clinical research certifications for investigators have you completed? Please check all that apply.

- [ ] Association of Clinical Research Professionals (Certified Principal Investigator)
- [ ] Other (Please specify)

4.4 Are there other principal investigators at your site who are neurologists and have experience conducting clinical trials? If so, please provide information about these individuals and their experience.

	PI Name	Clinical Trial Experience (Check all that apply)			Specify other disease(s)
		SMA	Other neuromuscular disease(s)	Other disease area(s)	
1		☐	☐	☐	
2		☐	☐	☐	
3		☐	☐	☐	
4		☐	☐	☐	
5		☐	☐	☐	

CLINICAL RESEARCH COORDINATORS (STUDY COORDINATORS)

VALIDATION Must be numeric

4.5 How many clinical research coordinators do you have available on staff that could support SMA research studies?

4.6 For coordinators who could support SMA trials, please provide information about the years of experience they have conducting the types of clinical trials below.

	Name	All Clinical Research (Years)	Neuromuscular Trials (Years)	SMA Trials (Years)
1				
2				
3				
4				
5				
6				
7				
8				
9				
10				

4.7 Please provide information about certifications held by coordinators at your site who could support SMA trials.

	Name	ACRP Certification	SOCRA Certification	Other Certifications & Year(s) Completed
1		☐	☐	
2		☐	☐	
3		☐	☐	
4		☐	☐	
5		☐	☐	
6		☐	☐	
7		☐	☐	
8		☐	☐	
9		☐	☐	
10		☐	☐	

VALIDATION Must be numeric

4.8 How many studies at your site are actively enrolling and how many patients (on average) does each coordinator typically manage?

actively enrolling studies: ____

patients typically managed by each coordinator: ____

PHYSICAL THERAPISTS

VALIDATION Must be numeric

4.9 How many physical therapists do you have available on staff to support SMA research studies?

4.10 Does your site have at least one physical therapist with experience conducting outcome measures for SMA or other neuromuscular diseases in the following settings?

	In Clinical Evaluation	In Clinical Trials
SMA-specific motor function outcome measures	☐	☐
Outcome measures for other neuromuscular diseases	☐	☐

4.11 How many physical therapists/evaluators are typically assigned per each study/trial?

List number of evaluators assigned per each study/trial.

STAFF TRAINING PERTAINING TO THE RESPONSIBLE AND ETHICAL CONDUCT OF CLINICAL RESEARCH

4.12 Have all relevant staff involved in clinical trials received the following types of training and certifications?

Check all that apply.

- ☐ Human Subjects Research Training (CITI module or equivalent)
- ☐ Good Clinical Practices (GCP) Certification
- ☐ Process of writing and administering informed consent (PI/CRC)
- ☐ FDA Regulated Research (CITI Module or through an academic institution)
- ☐ Research With Minors (CITI Module/or through an academic institution)
- ☐ HIPAA Compliance Training
- ☐ Bloodborne Pathogens (CRC/as pertinent to role)
- ☐ IATA Dangerous Goods (for shipping of clinical samples) (CRC/as pertinent to role)
- ☐ Other - Please list below any relevant training and/or certification not listed above.

Please list any relevant training and/or certification not listed above.

To save and exit, click the "Save and Continue Later" option displayed in the gray bar at the bottom of the survey browser window. You will be prompted to enter your email address and will receive a unique link, which will allow you to complete your responses at a later time.

SECTION 5. INFORMATION ABOUT CLINICAL STAFF

VALIDATION Must be numeric

5.1 How many of each of these types of professionals do you have on staff?

Neurologists:

Cardiologists:

Orthopedic Surgeons:

Nurse Practitioners (NPs):

Pulmonologists:

Respiratory Therapists:

GI Specialist:

Nutritionist/Dieticians:

Physical Therapists:

Occupational Therapists:

Electrophysiologists:

Genetic Counselors:

Social Workers:

Coordinators

To save and exit, click the "Save and Continue Later" option displayed in the gray bar at the bottom of the survey browser window. You will be prompted to enter your email address and will receive a unique link, which will allow you to complete your responses at a later time.

SECTION 6. COORDINATION OF PATIENT CARE

6.1 Do you have a process for coordinating the care of research patients recruited at your site but whose medical home is outside your institution?

- ○ Yes
- ○ No

6.2 Please briefly describe how matters pertaining to research and care follow-up (e.g. incidental findings at a research visit, or updates on changes in health status) are communicated and coordinated at your site.

6.3 What resources does your clinic have to support patient care coordination?

- ☐ Dedicated care coordinator
- ☐ Other

Please describe other resources that your clinic has to support patient care coordination.

6.4 Do you have an established process for ensuring adherence to standard of care for SMA?

○ Yes ○ No

How do you ensure that your patients are receiving standard of care?

To save and exit, click the "Save and Continue Later" option displayed in the gray bar at the bottom of the survey browser window. You will be prompted to enter your email address and will receive a unique link, which will allow you to complete your responses at a later time.

SECTION 7. ADDITIONAL QUESTIONS REGARDING CLINICAL TRIAL OPERATIONS

7.1 Does your clinical research unit conduct regular meetings?

○ Yes (please indicate the frequency) ○ No

7.2 Do you have a centralized IRB?

○ Yes

○ No (please indicate with which local IRB you intend to work)

7.3 Do you have an institutional biosafety committee?

- [ ] Yes
- [ ] No

7.4 Do you have a well-documented informed consent process?

- [ ] Yes
- [ ] No

7.5 Do you have an established and well-documented process for ensuring adherence to the study protocol?

- [ ] Yes
- [ ] No

Please provide a high level overview of your process.

7.6 Do you have an established and well-documented approach to PI oversight, in particular for addressing serious adverse events and managing records?

- [ ] Yes
- [ ] No

7.7 Please describe your site's storage capabilities for investigational products and biological samples (e.g. freezers, sample chain of custody, access restrictions, etc.).

LOGIC Show/hide trigger exists.

7.8 Have you or your practice been inspected by the FDA or a similar regulatory agency in the past five years?

○ Yes ○ No

LOGIC Hidden unless: Question "7.8 Have you or your practice been inspected by the FDA or a similar regulatory agency in the past five years?
" is one of the following answers ("Yes")

7.8.1 If yes, please specify the year of inspection and the outcome.

LOGIC Show/hide trigger exists.

7.9 Has your site ever been closed by a sponsor?

- ○ Yes
- ○ No

LOGIC Hidden by default Hidden unless: Question "7.9 Has your site ever been closed by a sponsor?
" is one of the following answers ("Yes")

7.9.1 Why was your site closed by a sponsor?

Check all that apply.

- ☐ Enrollment issues
- ☐ Conduct Issues
- ☐ Other (Please describe)

Additional issues leading to site closure

To save and exit, click the "Save and Continue Later" option displayed in the gray bar at the bottom of the survey browser window. You will be prompted to enter your email address and will receive a unique link, which will allow you to complete your responses at a later time.

SECTION 8. READINESS TO CONDUCT FUTURE RESEARCH & ADDITIONAL COMMENTS

8.1 Please describe any additional capabilities at your site or experience from other neuromuscular clinical trials that would support your ability to perform SMA research studies.

8.2 Are there any existing challenges, gaps, or concerns that would prevent you from conducting new clinical trials in SMA immediately or in the near future?

○ Yes ○ No

Please describe.

8.3 How could the Cure SMA readiness program best help you to meet your goal of becoming an SMA clinical trial site?

End of Survey

You have reached the end of the survey. If you believe you have reached this page in error, please contact **Fatou.Sarr@dbr.com**.

Thank You!

We appreciate your interest in helping to meet the needs of SMA patients and trial sponsors by participating in this process. We will be in touch soon about next steps.
