# Supplementary material for: The SMA Clinical Trial Readiness Program: creation and evaluation of a program to enhance SMA trial readiness in the United States
Source: Orphanet J Rare Dis. 2020 May 22;15:118. doi: 10.1186/s13023-020-01387-8 (PMC7564894; doi:10.1186/s13023-020-01387-8)
Supplement: Supplementary file 5 — Additional file 5. Site readiness checklist. [file 13023_2020_1387_MOESM5_ESM.pdf]

Sample Site Readiness Checklist		
**Minimum Criteria**	Clinical research infrastructure	✔
	Seeing SMA Patients for research or care	✔
**Site Research Capabilities and Experience**	Dedicated Clinical Research Unit	✔
	Clinical trial experience (any)	✔
	Neuromuscular clinical trial experience	✔
	Conducting SMA research studies (not clinical trials)	
	Active enrollment for SMA research studies (not clinical trials)	
**Patient Population**	Children	✔
	Adults	✔
**Principal Investigator Experience**	SMA clinical trial experience	✔
	Neuromuscular clinical trial experience (not SMA)	✔
	Certified Principal Investigator	
**Clinical Research Coordinator Experience**	SMA clinical trial experience	✔
	Neuromuscular clinical trial experience (not SMA)	
	Coordinator(s) has completed ACRP CRC or SOCRA certification	✔
**Physical Therapist Experience**	SMA-specific motor function outcome measures (clinical evaluation)	✔
	SMA-specific motor function outcome measures (clinical trials)	
	Neuromuscular disease outcome measures (not SMA)	✔
	Completed reliability training for motor function outcome measures	✔
**Staff Training Related to Conduct of Clinical Research**	Staff involved in clinical research have conducted all or majority of the listed training programs	✔
**Clinical Trial Operations**	Centralized IRB	
	Local IRB	✔
	Well-documented informed consent process	✔
	Established, well-documented approach for ensuring adherence to study protocol	✔
	Established and well-documented approach to PI oversight	✔
**Site Readiness (Site Self-Assessment)**	Does not have existing gaps/concerns that would prevent the site from conducting new clinical trials in SMA immediately or in the near future.	✔
